# TDP-43 proteinopathy alters the ribosome association of multiple mRNAs including the glypican Dally-like protein (Dlp)/GPC6

**DOI:** 10.1186/s40478-021-01148-z

**Published:** 2021-03-24

**Authors:** Erik M. Lehmkuhl, Suvithanandhini Loganathan, Eric Alsop, Alexander D. Blythe, Tina Kovalik, Nicholas P. Mortimore, Dianne Barrameda, Chuol Kueth, Randall J. Eck, Bhavani B. Siddegowda, Archi Joardar, Hannah Ball, Maria E. Macias, Robert Bowser, Kendall Van Keuren-Jensen, Daniela C. Zarnescu

**Affiliations:** 1 Department of Cellular and Molecular Biology, University of Arizona, 1007 E. Lowell St, LSS RM 548A, Tucson, AZ 85721 USA; 2 Translational Genomics Research Institute, 445 N 5th St, Phoenix, AZ 85004 USA; 3 Department of Neurobiology, Barrow Neurological Institute, 350 W Thomas Rd, Phoenix, AZ 85013 USA; 4 Department of Neuroscience, University of Arizona, 1040 4th St, Tucson, AZ 85721 USA; 5 Department of Neurology, University of Arizona, 1501 N Campbell Ave, Tucson, AZ 85724 USA

**Keywords:** ALS, TDP-43, Translation, Motor neuron, Neuromuscular junction, Glypican, Wnt signaling, Drosophila

## Abstract

Amyotrophic lateral sclerosis (ALS) is a genetically heterogeneous neurodegenerative disease in which 97% of patients exhibit cytoplasmic aggregates containing the RNA binding protein TDP-43. Using tagged ribosome affinity purifications in *Drosophila* models of TDP-43 proteinopathy, we identified TDP-43 dependent translational alterations in motor neurons impacting the spliceosome, pentose phosphate and oxidative phosphorylation pathways. A subset of the mRNAs with altered ribosome association are also enriched in TDP-43 complexes suggesting that they may be direct targets. Among these, *dlp* mRNA, which encodes the glypican Dally like protein (Dlp)/GPC6, a wingless (Wg/Wnt) signaling regulator is insolubilized both in flies and patient tissues with TDP-43 pathology. While Dlp/GPC6 forms puncta in the *Drosophila* neuropil and ALS spinal cords, it is reduced at the neuromuscular synapse in flies suggesting compartment specific effects of TDP-43 proteinopathy. These findings together with genetic interaction data show that Dlp/GPC6 is a novel, physiologically relevant target of TDP-43 proteinopathy.

**Supplementary Information:**

The online version contains supplementary material available at 10.1186/s40478-021-01148-z.

## Introduction

Amyotrophic lateral sclerosis (ALS) is a neurodegenerative disorder characterized by the progressive loss of motor neuron function, culminating in death due to respiratory failure [32, 79]. Mutations in several dozen genes including SOD1 [87], TDP-43 [45, 98], and C9orf72 [19, 84] have been implicated in disease pathogenesis, however for > 79% of patients the cause of disease remains unknown [52], suggesting that yet-to-be-uncovered mechanisms significantly contribute to the majority of ALS cases. At the cellular level, several processes have been linked to ALS including neuroinflammatory response [63], cellular metabolism [22, 106], RNA processing, axonal transport and protein homeostasis [105]. A hallmark of ALS is the finding that > 97% of patient spinal cord motor neurons exhibit cytoplasmic puncta containing the RNA binding protein TDP-43 (TAR DNA Binding Protein 43) [59, 77]. Together with reports that 2–3% of ALS patients harbor TDP-43 mutations [45, 77, 98, 109], these findings suggest a key role for TDP-43 in ALS pathogenesis regardless of etiology.

TDP-43 is a nucleo-cytoplasmic shuttling, DNA/RNA binding protein which is predominantly localized to the nucleus where it regulates transcription [101] and splicing [26, 108]. In the cytoplasm, TDP-43 regulates stress granule dynamics [21, 25, 46, 72] as well as axonal and dendritic mRNA localization and translation [1, 12, 15]. In disease, TDP-43 is depleted from the nucleus, causing splicing defects, derepression of cryptic exons [57] and increased retrotransposon expression [49, 61, 75, 103]. TDP-43 has been shown to induce toxicity through both nuclear loss-of-function and cytoplasmic gain-of-function mechanisms [93, 110, 113]. When mislocalized to the cytoplasm, TDP-43 associates with a plethora of RNA containing complexes including stress and transport granules [60, 89, 111], as well as protein complexes devoid of RNA [31, 69]. TDP-43 has also been shown to influence the translation of specific mRNAs, both as a negative and a positive regulator [12, 14, 15, 67, 76]. Taken together these findings suggest a complex role for TDP-43 in translation regulation, which has been linked to the formation of TDP-43 cytoplasmic puncta in disease and altered protein expression and/or localization of its mRNA targets.

A potential mechanism by which TDP-43 cytoplasmic inclusions dysregulate translation is by altering the ribosomes’ access to mRNAs, as suggested by the “ribostasis hypothesis” [83]. Further substantiating this model are recent reports of TDP-43 mediated translation inhibition of mRNAs, including *futsch* and *hsc70-4* mRNAs (in axons) [14, 15] and *Rac1* mRNA (in dendrites) [12], all of which were identified through candidate approaches. However, the relationship between TDP-43 associated mRNAs and translation in disease has not yet been examined through an unbiased approach. Here we combined RNA immunoprecipitations (RIP) and translating ribosome affinity purification (TRAP) to identify mRNAs that simultaneously satisfy two criteria: 1) are enriched in TDP-43 complexes and thus have the potential to be regulated by TDP-43, and 2) their association with ribosomes is altered in the context of *Drosophila* models of TDP-43 proteinopathy, consistent with an effect on translation. RIP experiments uncovered candidate mRNA targets linked to neuromuscular junction development and synaptic growth. TRAP uncovered mitochondrial metabolism, proteostasis and wingless signaling as significant components of the “normal” motor neuron translatome. When compared to controls, TDP-43 proteinopathy was found to significantly alter the ribosome association of multiple mRNAs whose cognate proteins play roles in splicing, purine metabolism and mitochondrial electron transport among others.

Here we identify Dally-like protein (Dlp), a glypican-type heparan sulfate proteoglycan (HSPG) that has been described as a regulator of wingless (Wg/Wnt) [120] and Liprin alpha receptor (LAR) mediated signaling at the neuromuscular junction [100] as a candidate target of TDP-43 mediated translation inhibition. We show that *dlp* mRNA is enriched in TDP-43 complexes and sequestered in insoluble aggregates, consistent with the ribostasis hypothesis. Consistent with this, we find that Dlp protein is significantly reduced at the neuromuscular junction while steady state levels of *dlp* transcript remain unchanged, further supporting the possibility of translation inhibition in axons and/or at synapses. Surprisingly, within the ventral nerve cord neuropil, Dlp accumulates in puncta, suggesting that TDP-43 proteinopathy affects Dlp in a compartment specific manner. Dlp depletion at the NMJ, but not puncta formation, was also observed with endogenous TDP-43 knockdown suggesting that some Dlp phenotypes are the result of loss of nuclear TDP-43 function while others are the results of toxic cytoplasmic gain of function. Genetic interaction experiments show that *dlp* overexpression mitigates TDP-43 dependent locomotor deficits, consistent with the notion that Dlp mediates aspects of TDP-43 proteinopathy in vivo. Lastly, GPC6 protein, a human homolog of Dlp, exhibits aggregate-like accumulations while GPC6 mRNA is enriched in insoluble fractions derived from ALS patient spinal cords, mirroring the findings from Drosophila motor neurons. Together, these findings highlight key pathways altered in the motor neuron translatome in the context of TDP-43 proteinopathy and support the notion that altered expression of the glypican Dlp/GPC6 contributes to motor neuron degeneration.

## Materials and methods

### Drosophila genetics

*w*^*1118*^*; UAS-TDP-43*^*WT*^*-YFP* and *w*^*1118*^*; UAS-TDP-43*^*G298S*^*-YFP* were previously described [23, 24]. *Drosophila* harboring UAS-RpL10 GFP were obtained from Herman Dierick [107]. *w*^*1118*^*; UAS-TDP-43*^*WT*^* UAS-RpL10-GFP/CyO* and *w*^*1118*^*; UAS-RpL10-GFP/CyO; UAS-TDP-43*^*G298S*^*/TM6B* were generated with *w*^*1118*^*; UAS-TDP-43*^*WT*^ [86] and *w*^*1118*^*; UAS-TDP-43*^*G298S*^ [44] using standard genetic approaches. *w*^*1118*^*; UAS-TBPH*^*RNAi*^ was generated by recombining *y*[1]* v*[1]*; P{y[*+ *t7.7] v[*+ *t1.8]* = *TRiP.HMS01846}fCRattP40* (VDRC v38377) and *y*[1]* v*[1]*; P{y[*+ *t7.7] v[*+ *t1.8]* = *TRiP.HMS01848}attP40* (VDRC v38379). These lines were crossed with the D42 GAL4 driver [37] to achieve motor neuron specific expression. For *dlp* overexpression we used *w*^*1118*^*; P{w[*+ *mC]* = *UAS-dlp.WT}3* (Bloomington Stock #9160) and for RNAi knock-down we used *y*[1]* v*[1]*; P{y[*+ *t7.7] v[*+ *t1.8]* = *TRiP.GLC01658}attP40* (Bloomington Stock #50540). As a control for the RNAi experiments we used *y*[1]* v*[1]*; P{y[*+ *t7.7]* = *CaryP}attP40* (Bloomington Stock #36,304). CRISPR TDP-43 knock-in models, TDP-43^WT^ and TDP-43^G294A^ in which the endogenous Drosophila TDP-43 (TBPH) had been replaced by human TDP-43 (WT or G294A) were previously described [9] and kindly provided by David Morton. For Fz2 overexpression we used w[*]; P{w[+ *mC]* = *UAS-fz2-2}16/TM6B, Tb[* +*] (*Bloomington Stock #41794. For Fz2 knockdown we used *y*[1] sc[*] v[1]* sev*[21]*;P{y[*+ *t7.7]v[*+ *t1.8]* = *TRiP.HMS05675}attP40* (Bloomington Stock #67863). Expression of dominant negative Fz2 was achieved with w[*]; P{w[+ *mC]* = *UAS-fz2(ECD)-GPI}3* (Bloomington Stock #44221). For G6PD overexpression, we utilized *w*^*1118*^*; UAS-G6PD* [54], kindly provided by William Orr.

### RNA immunoprecipitations (RIP)

100 third instar larvae expressing TDP-43^WT^-YFP or TDP-43^G298S^-YFP were collected and flash frozen in liquid nitrogen. Frozen larvae were homogenized in lysis buffer (100 mM HEPES Buffer pH 8.0, 1% Triton X-100, 200 mM NaCl, 30 mM EDTA, 350 mM Sucrose, 10% Glycerol, 1 mg/mL Heparin, 1 mM DTT, protease inhibitors (Millipore Sigma 11,873,580,001) and RNAsin Plus 400 units/ml (Fischer Scientific PRN2615). Lysates were centrifuged at 10,000 × g for 10 min and then pre-cleared with magnetic beads (Dynabeads Protein A, Thermofisher Scientific 10001D) for 1 h at 4 °C. Magnetic beads bound to chicken anti-GFP antibody (Life tech A-11122) were added to the supernatant followed by rotation at 4 ºC for 2 h. Next, beads were washed 3 times with a sugar-rich buffer (100 mM HEPES Buffer pH 8.0, 1% Triton X-100, 200 mM NaCl, 30 mM EDTA, 350 mM Sucrose, 10%, Glycerol, 1 mg/mL Heparin, 1 mM DTT, RNAsin Plus 400 u/ml) and 2 times with a low-density buffer (100 mM HEPES Buffer pH 8.0, 1% Triton X-100, 200 mM NaCl, 30 mM EDTA,1 mg/mL Heparin, 1 mM DTT, RNAsin Plus 400 units/ml (Fischer Scientific PRN2615) followed by resuspension in Qiagen RLT buffer (Qiagen 79,216) with 1% 2-mercaptoethanol (Sigma Aldrich M6250).

### Tagged ribosomes affinity purifications (TRAP)

RNA associated with ribosomes was immunoprecipitated using a protocol similar to RIP (see above) except that the genotypes were *w*^*1118*^*; UAS-RpL10-GFP/CyO*, *w*^*1118*^*; UAS-TDP-43*^*WT*^* UAS-RpL10-GFP/CyO* and *w*^*1118*^*; UAS-RpL10-GFP/CyO; UAS-TDP-43*^*G298S*^*/TM6B Hu Tb* and the buffers for the immunoprecipitation of RpL10-GFP also included 100 μg/mL cycloheximide.

### RNA isolation

RNA was isolated from the ventral cord lysates, whole larvae inputs, or immunoprecipitations using a Qiagen RNeasy Mini Kit (Qiagen 74104). For fractionation experiments, dissected ventral nerve cords (VNCs) or neuromuscular junctions (NMJ), RNA was isolated using Trizol (Thermofisher Scientific 15596026). With both isolation methods, RNA was eluted into 20 μl of molecular grade water with quality and quantity determined by a nanodrop spectrophotometer, measuring absorbance at 260/280 nm.

### RNA Seq

Isolated RNA was prepared for RNA sequencing using the SMART-seq v4 Ultra Low Input RNA kit (Takara Bio USA 634888). The Nextera XT DNA Library Prep Kit (Illumina FC-131-1024) was used to tag, clean, and pool the samples with quality assayed by an Agilent 2100 Bioanalyzer. The cDNA libraries for RIP/TRAP were sequenced by the Beijing Genomics Institute using an Illumina HiSeq 4000 and the Illumina Nextera XT Kit (Illumina 15032350) with 100-bp paired end reads. Human fractionation RNA-seq were performed at the Translational Genomics Institute (TGen) using similar protocols.

### RNA-seq analysis

Reads were quality filtered to > Q30 then aligned to the Flybase FB2018_05 genome using MAFFT [85] with differential expression analysis conducted through Deseq2 [64]. In RIP experiments, 9812 and 9954 genes were detected in TDP-43^WT^ and TDP-43^G298S^ complexes, respectively. In TRAP experiments, 9711, 9347, and 9669 genes were detected in RpL10 GFP complexes in the control, TDP-43^WT^, and TDP-43^G298S^ models, respectively. To identify the “normal” motor neuron translatome we calculated the Log2FoldChange between RpL10 IP and RpL10 VNCs. Next, to identify alterations resulting caused by TDP-43 proteinopathy to the motor neuron translatomes, we subtracted the Log2FoldChange between RpL10 IP and RpL10 VNCs from the Log2FoldChange between TDP-43^WT^ or ^G298S^ RpL10 IP and TDP-43^WT^ or ^G298S^ RpL10 IP VNCs. Gene ontology (GO) analyses were conducted using David 6.8 [42, 43]. Human spinal cord transcriptomic data underwent Deseq2 analysis from counts provided by target ALS, detecting 33,843 genes. For the human fractionation RNA-seq data, reads were aligned to the NCBI GRCh38 genome with GENCODE 29 genome using STAR 2.5.3a [22] then underwent Deseq2 differential expression analysis detecting 24,090 genes. GO plot 1.0.2 [114], STRING 11.0 [102], and the R package “LPS” version 1.0.10 were used to generate bio-informatic figures.

### Fractionations

Fractionations of both overexpression and CRISPR Drosophila models were conducted as previously described [14]. In brief, 25 third instar larvae (for the overexpression models) or 1–2 days old 25 homozygous flies (for the CRISPR lines) were homogenized in Trizol (Thermofisher Scientific 15596026). Lysates were then centrifuged at 25,000×*g* for 30 min. The supernatant became the soluble fraction and the pellet was solubilized in urea buffer (30 mM Tris, 7 M Urea, 2 M Thiourea, 4% CHAPS, 1X Protease Inhibitor Cocktail (Millipore Sigma 11873580001), 0.5 mM PMSF, RNAsin Plus 400 units/ml (Fischer Scientific PRN2615), pH 8.5) to generate the urea/insoluble fraction. For human fractionation samples, post-mortem tissue (four spinal cord and two cerebellum samples as controls) was homogenized and subjected to the fractionation protocol described above.

### Immunofluorescence (*Drosophila* tissues)

Third instar ventral nerve cords were dissected as previously described [24]. In brief, 3rd instar larvae were placed in saline on a Sylgard dissection plate and the ventral nerve cord was dissected for further processing. Primary antibodies/stains used were 1:5 anti-Dlp (antibody 13G8, developed by Phil Beachy, obtained from the Developmental Studies Hybridoma Bank, created by the NICHD of the NIH and maintained at The University of Iowa, Department of Biology, Iowa City, IA 52,242, targets amino acids V523 to Q702) and 1:200 anti-GFP (Rockland 600-102-215). DNA was visualized using Hoechst (1:10,000, Invitrogen H3570). Goat anti-mouse Alexa 568 (1:500 Thermofisher Scientific A-11004) was used as secondary antibody. Samples were imaged using a Zeiss LSM 880 inverted confocal microscope with a 40X oil lens. The number and cumulative area of Dlp puncta was quantified via manual counting and outlining of blinded VNC images.

Third instar larval NMJs were dissected as previously described [24]. In brief, 3rd instar larvae were pinned to Sylgard dissection plates and immersed in saline. The posterior end of the larvae was removed and the larvae were cut down the dorsal midline. Larvae were further pinned laterally to expose the lateral muscles innervated by motor neuron axons. Primary antibodies used were 1:5 anti-Dlp (antibody 13G8, developed by Phil Beachy, obtained from the Developmental Studies Hybridoma Bank, created by the NICHD of the NIH and maintained at The University of Iowa, Department of Biology, Iowa City, IA 52242, targets amino acids V523 to Q702), 1:100 anti-HRP Alexa 647 (Jackson Immuno-research 123-605-021). Goat anti-mouse Alexa Fluor 568 (1:500 Thermofisher Scientific A-11004) was used as secondary antibody. Samples were imaged using a Zeiss LSM 880 NLO upright confocal microscope with a 40X oil lens. Dlp intensity per area was quantified with NIH imageJ v1.52p within four terminal boutons defined by HRP staining. All images were blinded prior to being analyzed.

### Immunohistochemistry (human post-mortem spinal cords)

Paraffin-embedded post-mortem spinal cord tissue sections were obtained from the Barrow Neurological Institute ALS Tissue Bank and Target ALS Postmortem Tissue Core. All tissue samples were collected after informed consent from the subjects or by the subjects’ next of kin, complying with all relevant ethical regulations and approved by the appropriate Institutional Review Boards. Clinical neuropathological diagnoses were made by board certified neuropathologists according to consensus criteria. Subject demographics are listed in Additional file 4: Table S7-1. A total of 16 ALS and 7 controls (7 non-neurologic disease controls) were used in this study. All ALS cases have TDP-43 pathology while the non-neurologic disease controls lack TDP-43 pathology. Tissue sections were processed as previously described [5]. For immunohistochemistry (IHC) all tissue sections were deparaffinized, rehydrated and antigen retrieval performed using Target Antigen Retrieval Solution, pH 9.0 (Dako) in a steamer. Super Block (Scytek), supplemented with Avidin (Vector Labs) was used to block non-specific binding sites for 1 h. All sections were incubated overnight using rabbit polyclonal primary antibody GPC6 protein at 1:600 (Bioss bs-2177R) diluted in Super Block supplemented with Biotin (Vector Labs). A biotinylated goat anti-rabbit IgG (H + L) was used as secondary antibody (Vector Laboratories, BA-1000, 1:200) diluted in Super Block for 1 h. Immunostaining was visualized using the Vectastain Elite ABC reagent (Vector Labs) and Vector ImmPACT NovaRED peroxidase substrate kit (Vector Labs). Slides were counterstained with hematoxylin (Sigma Aldrich). Sections were visualized using an Olympus BX40 microscope. Images were blinded, then motor neurons were outlined and inverted in NIH ImageJ, which was also used to quantify GPC6 protein intensity relative to background. CellProfiler 3.1.9 [73] was used to quantify GPC6 puncta, defined between 3 to 13 pixels in diameter.

### qRT-PCR

All cDNA was synthesized using Fisher First Strand cDNA synthesis reagents (Thermofisher Scientific K1641). qPCR reactions were conducted in three biological replicates using Taqman Fast Advanced Master Mix (Thermofisher Scientific 4444556) and conducted on either an ABI 7100 or Analytik Jena 844-00504-4 qTOWER qPCR machine. *dlp* mRNA was quantified using Taqman assay (Thermofisher Scientific Dm01798597_m1) using *GPDH* (Thermofisher Scientific Dm01841185_m1) for normalization. Fold change was calculated using the standard ΔΔCT method [81].

### Locomotor assays

Third instar wandering larvae were placed on a grape juice agar plate. After being given 1 min to acclimate, the larvae were turned on the ventral side and timed until they uprighted themselves and began the first forward motion [15]. Data points outside of the median turning time ± 1.5IQR within each genotype were identified as outliers and excluded from statistical analyses.

### Fluorescent in situ hybridization (FISH)

FISH was performed using RNAscope (ACD Bio). A custom probe generated by ACD bio for dm-dlp and a control probe dm-Gapdh1 were used to detect *dlp* and *Gapdh1* in dissected VNC and NMJs as previously described [115]. Images were acquired using a Zeiss LSM 880 inverted confocal microscope with a 40X oil lens.

### Statistical analyses

For RNA seq analyses, significance was determined using the P adjusted value calculation embedded within Deseq2. All other statistical analyses were performed using GraphPad Prism 7.0 using two-tailed analyses. Normality was evaluated using the Kolmogorov–Smirnov test. All data shown are mean ± standard error of the mean.

## Results

### mRNAs enriched with TDP-43 in motor neurons encode proteins linked to neuronal and synaptic signaling

**Fig. 1:**
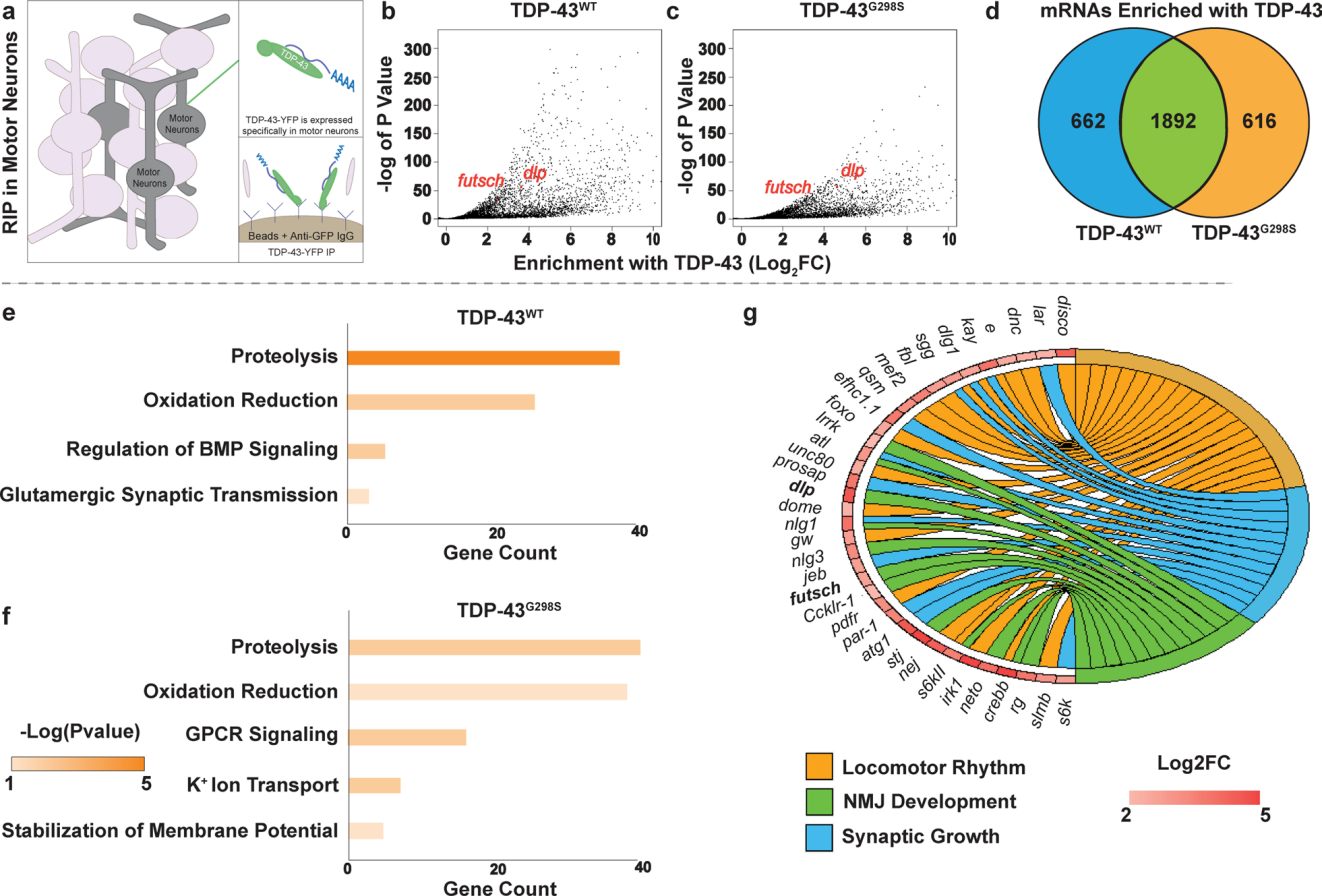
mRNAs Enriched with TDP-43 in A Drosophila Model of ALS. **a** Experimental schematic for RNA immunoprecipitations of human TDP-43, specifically from the motor neurons of third instar larvae. **b**, **c** Volcano plot displaying mRNAs enriched with TDP-43 in TDP-43^WT^ (**b**) and TDP-43^G298S^. All genes associated with TDP-43 (log2FC > 0) are displayed regardless of significance. **c**) proteinopathy, relative to transcript levels in the ventral nerve cord. **d–g **GO terms for TDP-43 enriched genes (log2FC > 2, *P*_adj_ < 0.05) either unique to TDP-43^WT^ (**e**) or TDP-43^G298S^ (**f**) or shared between genotypes (**g**)

TDP-43 has been shown to associate with a plethora of RNA targets and regulate various aspects of RNA processing including RNA localization and translation [1, 15, 76]. These findings suggest that cytoplasmic TDP-43, which is a hallmark of disease, has multiple opportunities to cause alterations in the motor neuron proteome and contribute to pathogenesis. To uncover these alterations, we defined TDP-43 dependent changes in the motor neuron translatome in vivo using *Drosophila* models of TDP-43 proteinopathy that recapitulate key aspects of the disease including locomotor defects, cytoplasmic aggregates and reduced lifespan [23, 24]. We hypothesized that while some of the translational alterations may be an indirect consequence of neurodegeneration, others are directly caused by TDP-43, possibly through mRNA association with, and sequestration into, TDP-43 cytoplasmic complexes. To distinguish between direct versus indirect effects on the translatome we set out to identify mRNAs that are both enriched with TDP-43 and translationally dysregulated in motor neurons in vivo using a combination of RNA immunoprecipitations (RIP) and Translating Ribosomes Affinity Purifications (TRAP) [39, 107] in the context of TDP-43 proteinopathy. To this end, we first used *Drosophila* larvae expressing human TDP-43 protein specifically in motor neurons via the GAL4-UAS system (D42 GAL4 > UAS-TDP-43-YFP) and performed immunoprecipitation experiments to pull down TDP-43 and associated mRNAs (RIP). The mRNAs associated with TDP-43 (WT or mutant G298S) were isolated and subjected to RNA sequencing (see Materials and Methods, and Fig. 1a). We have previously shown that TDP-43^WT^ and TDP-43^G298S^ are expressed at comparable levels [24], thus any differences between these transgenics indicate variant specific alterations rather than different expression levels. Bioinformatic analyses identified several mRNAs significantly enriched with TDP-43, as determined by comparing TDP-43 associated mRNAs in a complex with the transcriptome of dissected ventral nerve cords expressing the appropriate TDP-43 variant (TDP-43^WT or G298S^, see Fig. 1b,c). Of the approximately 9,900 transcripts detected in ventral nerve cords (Additional file 1: Table S1-1), 1,892 were enriched with, and shared by, both TDP-43 variants (Log2FC > 2, *P*_adj_ < 0.05). Additionally, about 25% of mRNAs enriched with TDP-43 were unique to TDP-43^WT^ or TDP-43^G298S^ (662 mRNAs enriched with TDP-43^WT^ and 616 mRNAs enriched with TDP-43^G298S^, respectively, see Fig. 1d) consistent with previous evidence that wild-type and mutant TDP-43 variants cause toxicity in part through distinct mechanisms (see also Fig. 1e, f) [14].

GO term analyses using David 6.8 [42, 43] of mRNAs enriched with both TDP-43^WT^ and TDP-43^G298S^ (Log2FC > 2, *P*_adj_ < 0.05) highlight neuronal pathways previously linked to TDP-43 such as NMJ development [33] and synaptic growth [23, 99] (Fig. 1e, g, Additional file 1: Table S1-2,3,4) and are consistent with published studies of TDP-43 interacting RNAs in a murine model of TDP-43 proteinopathy [91]. In addition, our analyses identified previously published mRNA targets of TDP-43 including *futsch* [15] (TDP-43^WT^, Log2FC = 2.44, *P*_adj_ = 8.07E−37; TDP-43^G298S^, Log2FC = 2.68, *P*_adj_ = 6.86 E−12) and *CSNK1E* [48] (TDP-43^WT^, Log2FC = 0.721, *P*_adj_ = 3.32E−3; TDP-43^G298S^, Log2FC = 0.847, *P*_adj_ = 3.08E−3). Furthermore, we found that of the 834 TDP-43 interacting mRNAs recently identified in murine neurons using TRIBE (targets of RNA-binding proteins identified by editing) [40] about 1/3 were also enriched in our *Drosophila* RIP data sets (33.4% for TDP-43^WT^, 34.9% for TDP-43^G298S^, Additional file 1: Table S1-5). In summary, our RIP experiments identify several mRNA candidate targets of TDP-43 in motor neurons, in vivo, a fraction of which have been previously identified in mammalian neurons [15, 40, 48] while others are novel; together they highlight links between TDP-43 proteinopathy and neuronal function, neuromuscular junction development and synaptic growth.

### TDP-43 proteinopathy induces a broad range of translational changes in motor neurons

**Fig. 2:**
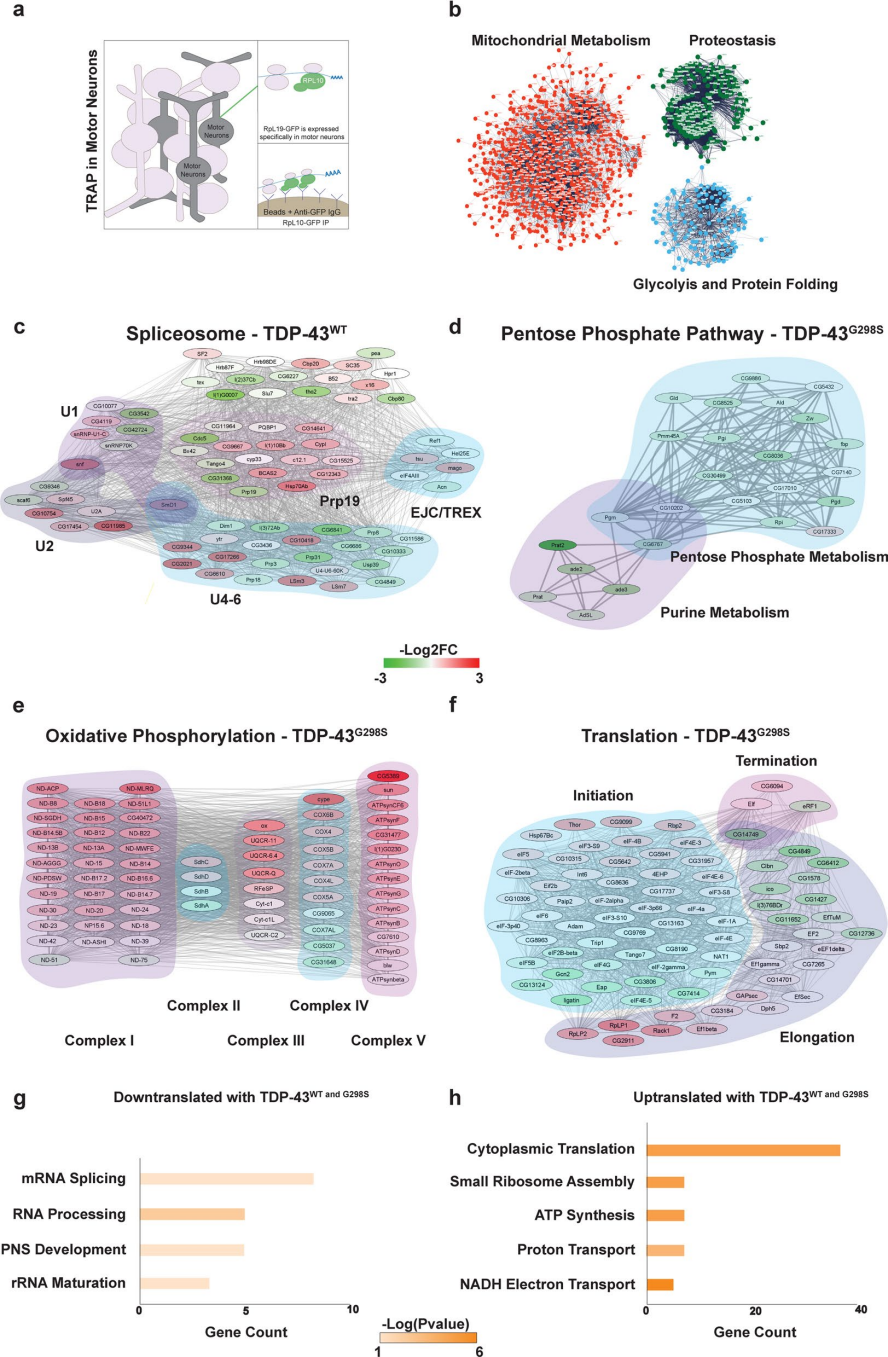
Translational Alterations Induced by TDP-43 Proteinopathy. **a** Experimental schematic for RNA immunoprecipitations of human TDP-43, specifically from the motor neurons of third instar larvae. **b** STRING clusters from genes in the top 10% of normalized counts associated with RpL10-GFP in w^1118^ larvae (control motor neuron translatomes). **c** Altered ribosome association of spliceosome genes in TDP-43^WT^ relative to the control. **d** Altered ribosome association of purine metabolism genes in TDP-43^G298S^ relative to control. **e** Altered ribosome association of oxidative phosphorylation genes in TDP-43^G298S^ relative to control. **f** Altered ribosome association of translation associated genes in TDP-43^G298S^ relative to control. **g**, **h** GO terms for RpL10 depleted genes (log2FC < −1) (**g**) and RpL10 enriched genes (log2FC > 1) (**h**) shared between both TDP-43^WT^ and TDP-43^G298S^ models

Next, to define the in vivo motor neuron translatome and identify changes in translation induced by TDP-43 proteinopathy (see Materials and Methods, and Fig. 2a) we conducted TRAP experiments [39, 107]. To this end, RpL10-GFP was expressed in motor neurons using D42 GAL4 either on its own, or together with TDP-43^WT^, or TDP-43^G298S^. Following ribosome immunoprecipitations we isolated total RNA and conducted RNA seq, detecting ~ 9,500 mRNAs (9,711 mRNAs for RpL10 controls, 9,347 for TDP-43^WT^ and 9,669 for TDP-43^G298S^, see Materials and Methods, Additional file 2: Table S2-1). We first defined the “normal” motor neuron translatome using the normalized gene counts associated with ribosome immunoprecipitations from RpL10-GFP controls (Additional file 2: Table S2-2). Using David 6.8 [42, 43], we determined that genes enriched in the top 10% of the motor neuron translatome normalized counts were related to proteostasis and energy metabolism (Fig. 2b, Additional file 2: Table S2-3), consistent with high levels of protein turnover [2] and energy demands in neurons [94].

To identify changes in translation caused by TDP-43 proteinopathy in motor neurons in vivo, we subsequently compared the mRNAs enriched with ribosomes precipitated from *Drosophila* larvae expressing RpL10-GFP TDP-43^WT^ or RpL10-GFP TDP-43^G298S^ to RpL10-GFP controls after normalizing to the transcriptomes of dissected ventral nerve cords of the appropriate genotype (RpL10-GFP TDP-43^WT^, RpL10 GFP TDP-43^G298S^ or RpL10-GFP). These comparisons identified several genes enriched with or depleted from motor neuron ribosomes in both of the ALS models relative to RpL10-GFP controls (ΔLog2FC > 1 or < −1). Subsequent GO term analyses using David 6.8 [42, 43] revealed that in the context of TDP-43^WT^, spliceosome components were translationally dysregulated (Fig. 2c, Additional file 2: Table S2-4), suggesting that TDP-43^WT^ overexpression causes pathway alterations previously associated with nuclear loss-of-function phenotypes [3, 18]. Interestingly, in the context of TDP-43^G298S^, we found significant alterations in neuronal metabolism (Additional file 2: Table S2-5), including purine processing leading into the pentose phosphate pathway (PPP), which provides a mechanism for countering oxidative stress via increased NADPH production [50]. PPP was found to be down-translated, consistent with our previous studies showing that glucose 6 phosphate dehydrogenase (G6PD), the rate limiting enzyme in PPP is altered in flies and patient tissues with TDP-43 pathology [70] (Fig. 2d). Further validating this finding, overexpression of G6PD mitigated TDP-43^G298S^ induced locomotor dysfunction (Supplemental Fig. 2-1a). Interestingly, oxidative phosphorylation genes encoding components of the electron transport chain showed both increased and reduced association with ribosomes (Fig. 2e). Recent findings of reduced Complex I activity would suggest that increased translation of Complex I components identified via TRAP may reflect a compensatory mechanism [22, 106, 117].

Interestingly, genes salient to translation exhibited altered translation (Fig. 2f). In both the TDP-43^WT^ and TDP-43^G298S^ models, ribosomal RNA maturation was down-regulated (Fig. 2g, Additional file 2: Table S2-6) while cytoplasmic translation components were up-regulated relative to the control (Fig. 2h, Additional file 2: Table S2-7). These findings are consistent with a previous study in which patient derived iPSC neurons exhibited a compensatory increase in global translation [104]. Further corroborating our findings with previous studies, we detected increased association with ribosomes for *CG6762*, the *Drosophila* ortholog of human SRXN1, (TDP-43^WT^, Log2FC = 0.673, *P*_adj_ = 2.79E−32; TDP-43^G298S^, Log2FC = 0.328, *P*_adj_ = 2.50E−51), one of 14 genes that exhibited increased translation in a human cell model of TDP-43^A315T^ proteinopathy [76]. Taken together, our ribosomal tagging experiments highlight complex changes in translation, some of which may be direct consequences of TDP-43 proteinopathy while others may reflect compensatory mechanisms.

### A fraction of TDP-43 associated mRNAs are altered in their association with ribosomes in the context of TDP-43 proteinopathy

**Fig. 3:**
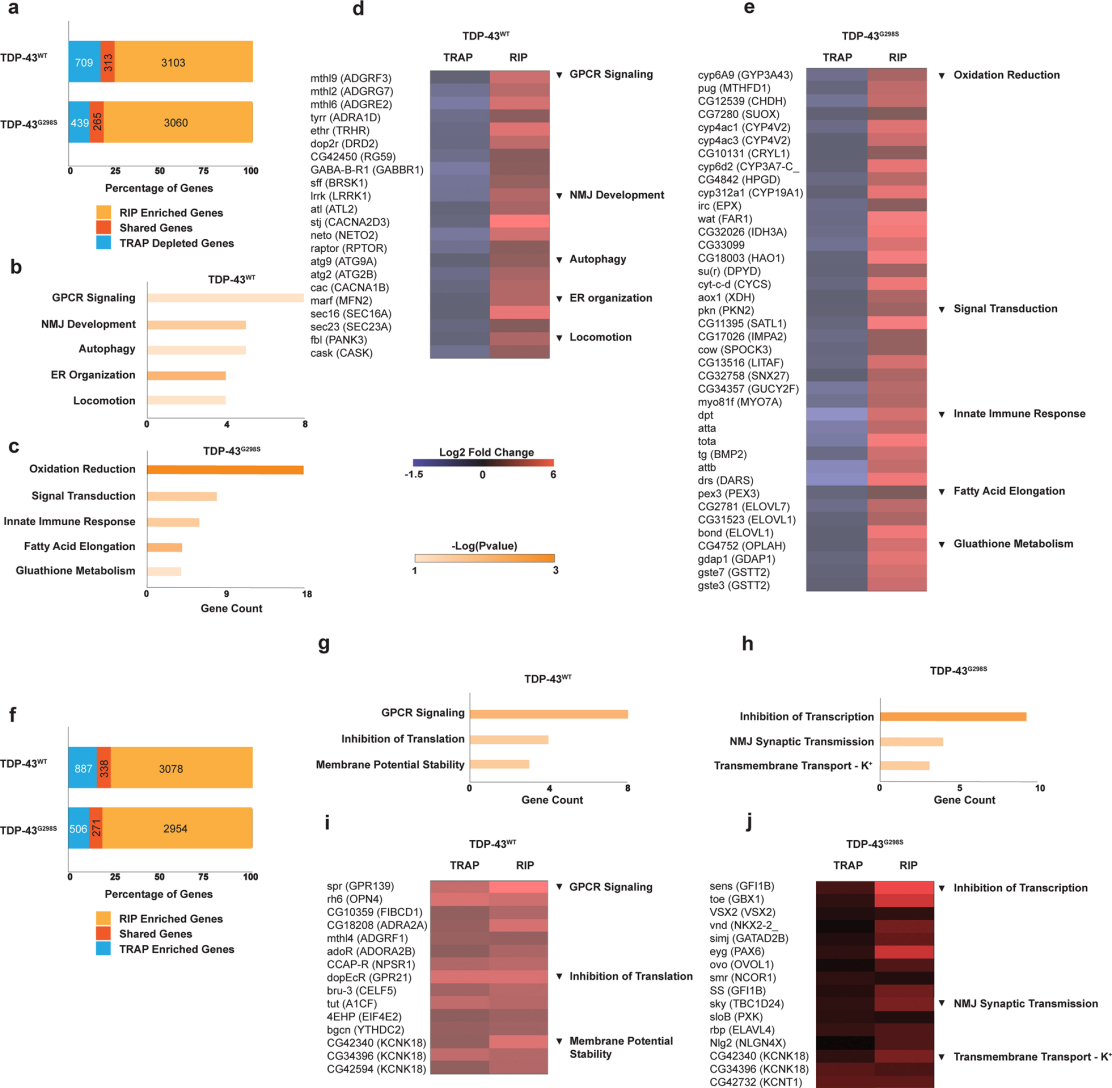
Direct Targets of TDP-43 Mediated Translation Regulation. **a** Overlap between genes enriched with TDP-43 (log2FC > 1, *P*_adj_ < 0.05) and depleted with RpL10 (log2FC < −1) relative to the w^1118^ control. **b**, **c** GO terms for TDP-43 enriched, RpL10 depleted genes.in TDP-43^WT^ (**b**) or TDP-43^G298S^ (**c**) models. **d**, **e** RpL10 depletion (TRAP) and TDP-43 enrichment (RIP) for genes constituting significant GO terms in TDP-43^WT^ (**d**) and TDP-43^G298S^ (**e**). **f** Overlap between genes enriched with TDP-43 (log2FC > 1, *P*_adj_ < 0.05) and enriched with RpL10 (log2FC > 1) relative to the w^1118^ control. **g**,** h** GO terms for TDP-43 enriched, RpL10 enriched genes.in TDP-43^WT^ (**g**) or TDP-43^G298S^ (**h**) models. **i, j** RpL10 enrichment (TRAP) and TDP-43 enrichment (RIP) for genes constituting significant GO terms in TDP-43^WT^ (**i**) and TDP-43^G298S^ (**j**). **d–e, i–j** Closest human orthologs identified using DIOPT are denoted in parentheses to the right of the fly gene names

To distinguish between translational changes that may be caused directly by TDP-43 versus compensatory alterations, we compared genes altered in their association with ribosomes to those enriched in TDP-43 complexes, in the context of TDP-43 proteinopathy. These analyses show that approximately 1/3 of the genes depleted from ribosomes were also enriched in TDP-43 complexes (Fig. 3a, Additional file 3: Table S3-1, 30.6% for TDP-43^WT^, 37.6% for TDP-43^G298S^) suggesting that these may be direct targets of translation inhibition. GO term analyses of these TDP-43 enriched, TRAP depleted genes using David 6.8 [42, 43] identified pathways that have previously been associated with ALS including GPCR signaling [28, 97], NMJ Development [28, 97], Autophagy [6, 116], ER Organization [112], Immune Response [65] and Oxidation Reduction [95] among others (Fig. 3b–e, 3: Table S3-2,3). Interestingly, a similar proportion (Fig. 3f, Additional file 3: Table S3-4, TDP-43^WT^ 27.6%, TDP-43^G298S^ 34.9%) of the genes enriched with ribosomes in the context of TDP-43 proteinopathy were also associated with TDP-43 complexes (Fig. 3f, Additional file 3: Table S3-4), consistent with previous finding that TDP-43 can also function as a positive regulator of translation [76]. GO term analyses identified pathways such as Membrane Potential Stability and Transmembrane Transport—K+ (Fig. 3g–j, Additional file 3: Table S3-4) comprising genes related to membrane excitability, a process known to be altered in ALS [74]. We note that although GO term analysis is useful to identify translational alterations affecting multiple components of the same pathway, the genes encompassed by these GO terms represent only a fraction of genes enriched and translationally dysregulated in the context of TDP-43 (Fig. 3a, f, 13.7% for TDP-43^WT^, 12.4% for TDP-43^G298S^). This suggests that in addition to specific pathways, a plethora of individual genes might represent salient targets of TDP-43 mediated translational inhibition or activation.

### *dally-like protein* (*dlp*) mRNA, a glypican involved in wingless (Wg/Wnt) signaling is sequestered in insoluble complexes in *Drosophila* models of TDP-43 proteinopathy

**Fig. 4:**
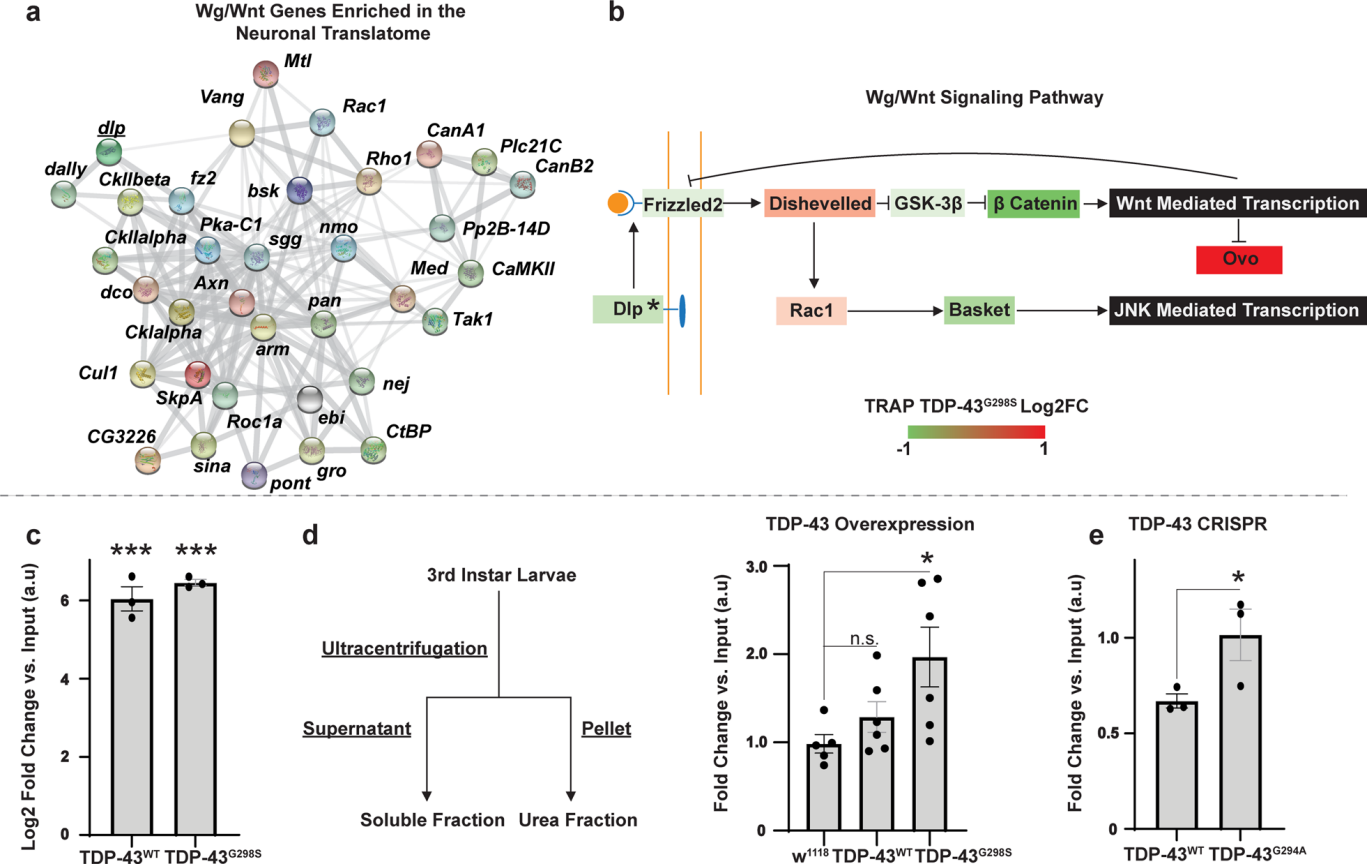
*dlp* mRNA, a Wg/Wnt interactor, is enriched in TDP-43 complexes and sequestered in insoluble fractions. **a** STRING diagram [102] depicting relationship between Wg/Wnt signaling genes in the top third of the TRAP control IP normalized counts (D42 > RpL10 GFP, see Materials and Methods). Line thickness between genes is correlated with the strength of the interaction as defined by STRING. **b** Altered ribosome association of a subset of Wnt signaling genes in TDP-43^G298S^ relative to control. **c** qPCR quantification of *dlp* mRNA enrichment with TDP-43 complexes in TDP-43^WT^ and TDP-43^G298S^ expressing larvae (D42 > TDP-43 WT or G298S). Enrichment in TDP-43 complexes is shown as arbitrary units (*a.u.*) after normalization to input. **d,e**. Schematic of fractionation experiments and qPCR quantification of *dlp* mRNA levels in the insoluble fraction (Urea fraction) of TDP-43^WT^ or TDP-43^G298S^ expressing larvae relative to w^1118^ controls. N = (w^1118^ = 5, other = 6) (**d**), and CRISPR-TDP-43^G294A^ (relative to CRISPR-TDP-43^WT^, N = 3). Significance was determined using the Holm Sidak’s multiple comparisons test (**b**, **d**) or Student’s *T* Test (**e**). *P_value_ < 0.05, ***P_value_ < 0.001. Error bars represent SEM

Additional analyses of the “normal” motor neuron translatome using STRING [102] identified an enrichment in Wg/Wnt signaling genes (Fig. 4a), with the top 5% of genes translated in motor neurons containing Wg/Wnt signaling genes at a rate 61% greater than that of the entire data set. This is consistent with the established role of Wg/Wnt signaling in motor neurons and at the neuromuscular junction [47]. Interestingly, Wg/Wnt signaling has been shown to be dysregulated in ALS [11, 34, 35], although its mechanisms remain poorly understood, in part due to the complexity of Wg/Wnt signaling and cross-talk with other pathways. Given these observations, we queried the pathway for candidate targets of TDP-43 mediated translational inhibition and found *dlp* mRNA to be enriched in TDP-43 complexes (TDP-43^WT^, Log2FC = 3.62, *P*_adj_ = 8.32E−57; TDP-43^G298S^, Log2FC = 4.63, *P*_adj_ = 2.47E−57) and significantly depleted from ribosomes in the context of TDP-43 proteinopathy (TDP-43^WT^, Log2FC = −1.03, *P*_adj_ = 0.027; TDP-43^G298S^, Log2FC = −0.85, *P*_adj_ = 0.012, see also Additional file 5: Table S4-1). *dlp* encodes a glypican, a member of the heparan sulfate proteoglycan (HSPG) family, localized to the plasma membrane via a GPI anchor or secreted in the extracellular matrix. HSPGs consist of a protein core and several heparan sulfate (HS) glycosaminoglycan (GAG) linear polysaccharide chains which exhibit complex sulfation patterns that dictate the ability of the HSPGs to interact with ligands such as *wingless* [120]. In *Drosophila*, Dlp was shown to function as a co-factor and competitive inhibitor for binding to the Wg/Wnt pathway receptor Frizzled 2 [120]. Interestingly, additional components of Wg/Wnt signaling exhibit altered ribosomal association including *frizzled 2* mRNA (TDP-43^WT^, Log2FC = −1.00, *P*_adj_ = 0.026; TDP-43^G298S^, Log2FC = −0.56, *P*_adj_ = 0.027) and *ovo/shavenbaby* mRNA (TDP-43^WT^, Log2FC = 2.74, *P*_adj_ = 4.38E−8; TDP-43^G298S^, Log2FC = 3.02, *P*_adj_ = 2.15E−21) (Fig. 4b), further substantiating the possibility that TDP-43 proteinopathy affects this signaling pathway.

To validate *dlp* mRNA as a target of TDP-43 in motor neurons, we first performed TDP-43 immunoprecipitations from *Drosophila* larvae expressing either TDP-43^WT^ or TDP-43^G298S^ in motor neurons then isolated the associated RNA and used RT-qPCR to amplify the *dlp* transcript. These experiments showed that indeed, *dlp* mRNA was enriched with both TDP-43^WT^ (Log2FC = 6.11, *P*_value_ = 5.54E−7) and TDP-43^G298S^ (Log2FC = 6.46, *P*_value_ = 5.54E−7) relative to input (Fig. 4c), confirming the RIP mRNA seq data (Fig. 1a–d).

Next, to determine whether *dlp* mRNA is insolubilized as predicted by the ribostasis hypothesis, we conducted subcellular fractionations and quantified *dlp* mRNA in the soluble and insoluble/urea fractions from third instar larvae expressing TDP-43 in motor neurons. These experiments showed that *dlp* mRNA is significantly insolubilized in the context of TDP-43^G298S^ (Fold change = 1.99, *P*_value_ = 0.0256, Fig. 4d) but not in the context of TDP-43^WT^ overexpression. Whether *dlp* mRNA is insolubilized in the same complex as TDP-43^G298S^ or in a different complex remains to be determined.

Further substantiating this finding, *dlp* mRNA was insolubilized in a TDP-43^G294A^ (Fold change = 2.51, *P*_value_ = 0.034, Fig. 4e) but not in the TDP-43^WT^ CRISPR fly model of ALS in which the endogenous *Drosophila* TDP-43 (TBPH) gene has been replaced with human TDP-43 [9]. Taken together these results indicate that *dlp* mRNA is a novel TDP-43 candidate target that becomes insolubilized in the context of TDP-43 proteinopathy and are consistent with previous reports of Wg/Wnt alterations in disease.

### Dlp protein levels are altered in the neuromuscular system in the context of TDP-43 proteinopathy

**Fig. 5:**
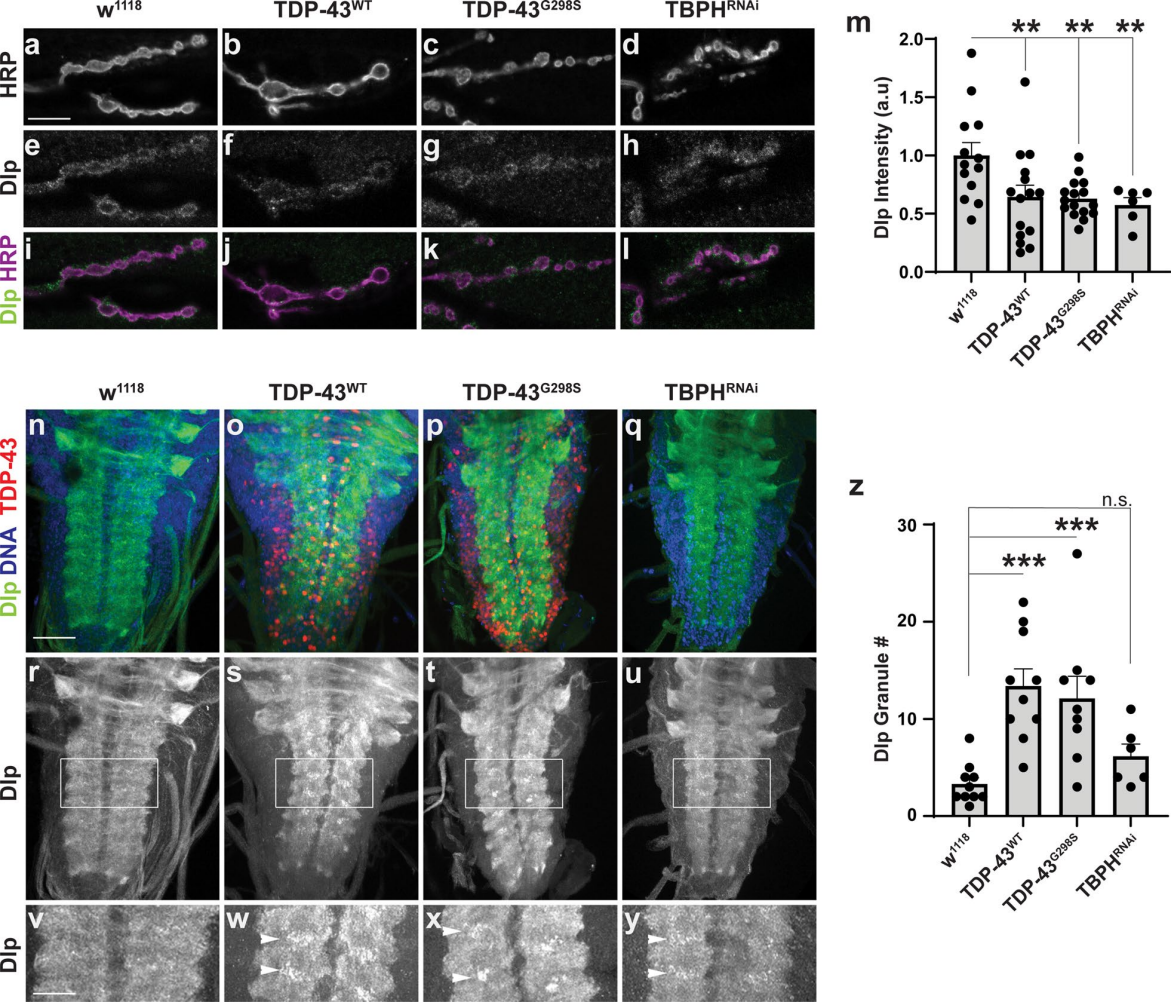
Dlp protein expression is altered by TDP-43 proteinopathy. **a–l** Representative images of w^1118^, TDP-43^WT^, TDP-43^G298S^ and TBPH^RNAi^ NMJs stained for Dlp and HRP. **m** Quantification of Dlp protein relative to bouton area in terminal boutons. N = 13 for w^1118^, 15 for TDP-43^WT^, 16 for TDP-43^G298S^, 6 for TBPH^RNAi^. **n–y** Representative images of w^1118^, TDP-43^WT^, TDP-43^G298S^ and TBPH^RNAi^ VNCs stained for Dlp, DNA and TDP-43 (**z**)**.** Quantification of Dlp granule number in the neuropil. N = 10 for w^1118^, 10 for TDP-43^WT^, 9 for TDP-43^G298S^, 6 for TBPH^RNAi^. Genotypes and stainings, as indicated. Scale bars: **a** 10 μm**, n** 50 μm. **v** 22 μm. Significance determined using the Holm Sidak’s multiple comparison test. *P_value_ < 0.05, **P_value_ < 0.01, ***P_value_ < 0.001. Error bars represent SEM

A possible outcome of *dlp* mRNA enrichment with TDP-43 complexes, insolubilization and decreased association with ribosomes is inhibition of Dlp protein synthesis. To test the impact of TDP-43 proteinopathy on Dlp protein expression we conducted immunofluorescence experiments in the larval neuromuscular system. We examined Dlp expression at the neuromuscular junction (NMJ) and the ventral nerve cord (VNC), which comprises motor neuron cell bodies and compactly packaged neurites, particularly dendrites [29] (Fig. 5a–z). These experiments showed that overexpression of TDP-43^WT^ or TDP-43^G298S^, and RNAi knock-down of TBPH (TBPH^RNAi^) are sufficient to deplete Dlp protein within NMJ boutons relative to the w^1118^ control (TDP-43^WT^, decreased 35.7%, *P*_value_ = 0.00920; TDP-43^G298S^, decreased 37.2%, *P*_value_ = 0.00860; TBPH^RNAi^, decreased 42.5%, *P*_value_ = 0.00920; Fig. 5m). While no major changes in Dlp expression were detected in the motor neuron cytoplasm within the VNC (data not shown), striking aggregate-like structures were observed in the neuropil when either TDP-43^WT^ or TDP-43^G298S^ were overexpressed (see Fig. 5n–y). Quantification shows that both TDP-43^WT^ and TDP-43^G298S^ proteinopathy cause a statistically significant increase in Dlp granule number (TDP-43^WT^, Fold Change = 4.99, *P*_value_ = 1.77E−4; TDP-43^G298S^, Fold Change = 4.24, *P*_value_ = 8.49E−4) and cumulative granule area relative to the w^1118^ controls. In contrast, TBPH^RNAi^ did not cause significant Dlp granularity phenotypes (TBPH^RNAI^, Fold Change = 2.03, *P*_value_ = 0.262, Fig. 5n–z and Additional file 5: Figure S5-1a).

These results indicate that TDP-43 proteinopathy causes altered Dlp protein expression, more specifically a reduction at synaptic terminals and aggregate like structures in the VNC neuropil. To investigate whether these alterations are post-transcriptional, as suggested by the RIP and TRAP experiments, we used fluorescence in situ hybridization (RNAScope) to examine *dlp* mRNA localization in the VNC and at the NMJ. Since we were not able to detect a specific signal, possibly due to low levels of expression (data not shown) we performed RT-qPCR experiments using dissected VNCs and larval NMJ preparations and found no significant changes in *dlp* mRNA levels between TDP-43 proteinopathy models and controls (Additional file 5: Figure S1b-c). Although the VNC and NMJ qPCR samples contain multiple cell types, which affects our ability to detect mRNA differences caused by TDP-43 overexpression specifically in motor neurons, these experiments collectively suggest that Dlp expression is likely regulated by TDP-43 post-transcriptionally, possibly through a combination of translation and axonal transport.

### *dlp* is a modifier of TDP-43 dependent locomotor deficits

**Fig. 6:**
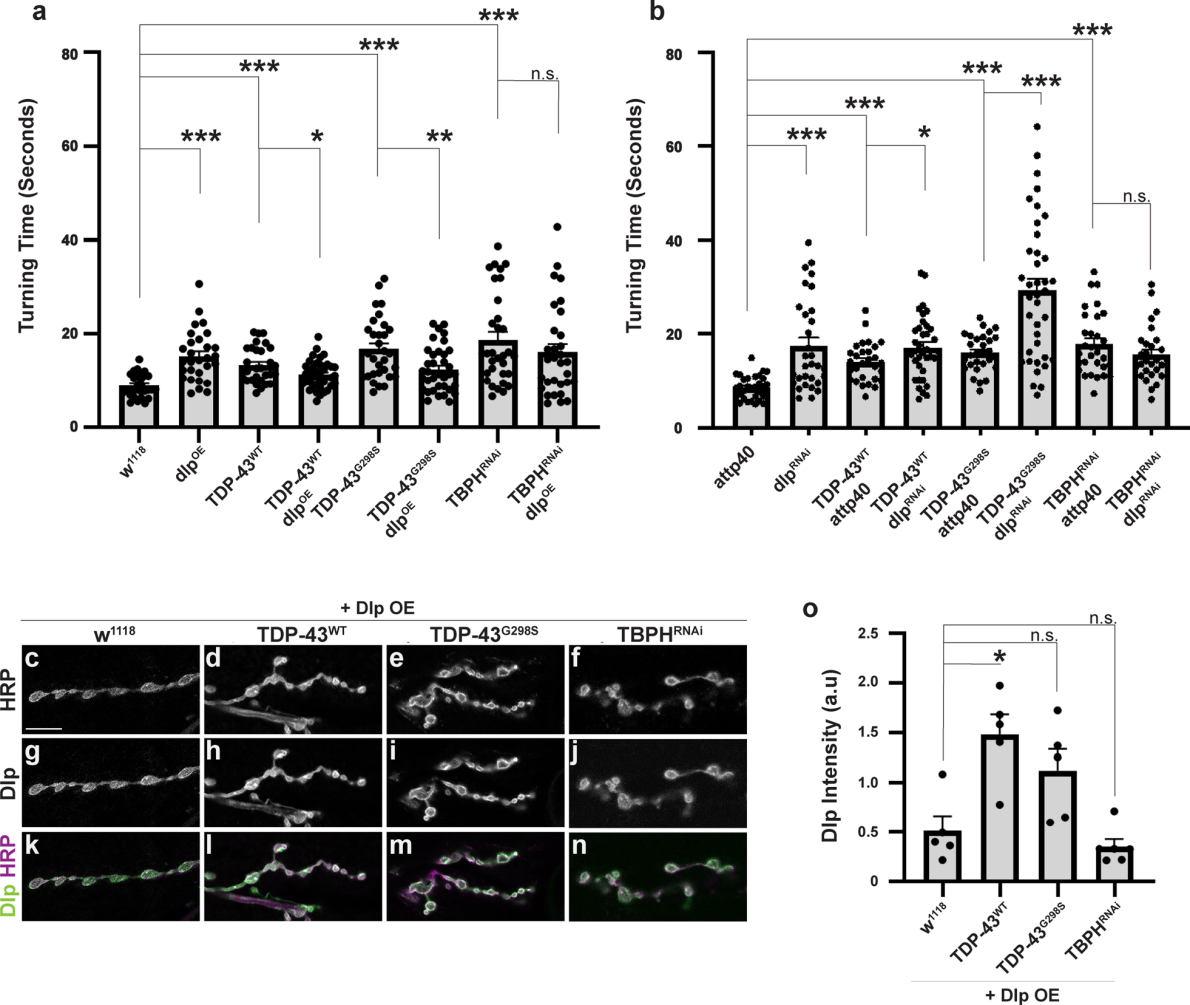
Altered *dlp* mRNA levels modify TDP-43 induced locomotion defects. **a** Larval turning times for *dlp* mRNA overexpression in the w^1118^ genetic background (control for OE experiments), TDP-43^WT^, TDP-43^G298S^ and TBPH^RNAi^. N = 29 for w^1118^, 29 for dlp^OE^, 30 for TDP-43^WT^, 37 for TDP-43^WT^ dlp^OE^, 31 for TDP-43^G298S^, 36 for TDP-43^G298S^ dlp^OE^, 32 for TBPH^RNAI^, 31 for TBPH^RNAi^ dlp^OE^. **b** Larval turning times for *dlp* RNAi in the attp40 genetic background (control for RNAi experiments), TDP-43^WT^, and TDP-43^G298S^. N = 30 for attp40, 31 for dlp^RNAi^, 29 for attp40 TDP-43^WT^, 37 for TDP-43^WT^ dlp^RNAi^, 29 for attp40 TDP-43^G298S^, 38 for TDP-43^G298S^ dlp^RNAi^, 30 for TBPH^RNAi^, 28 for TBPH^RNAi^ dlp^RNAi^. Significance determined using the MannU Whitney Test or Holm Sidak’s multiple comparison test. *P_value_ < 0.05, **P_value_ < 0.01, ***P_value_ < 0.001. **c–n** Representative images of *dlp*^*OE*^ NMJs in the context of w^1118^, TDP-43^WT^, TDP-43^G298S^ and TBPH^RNAi^ stained for Dlp and HRP. **o** Quantification of Dlp intensity in NMJ terminal boutons. N = 5 for w^1118^ dlp^OE^, 5 for TDP-43^WT^ dlp^OE^, 5 for TDP-43^G298S^ dlp^OE^, 6 for TBPH^RNAi^ dlp^OE^. Genotypes and stainings, as indicated. Scale bars: **c** 10 μm. *P_value_ < 0.05, **P_value_ < 0.01, ***P_value_ < 0.001. Error bars represent SEM

Next, to test whether Dlp is an effector of TDP-43 proteinopathy, we overexpressed *dlp* using the motor neuron specific driver D42 GAL4 on its own and in the context of TDP-43^WT^ or TDP-43^G298S^ and measured its effect on TDP-43 induced locomotor defects. Using larval turning assays to measure locomotor function we found that although *dlp* overexpression (*dlp*^OE^) on its own resulted in a higher turning time (15.3 ± 1.01 s compared to w^1118^ controls 8.95 ± 0.46 s, *P*_value_ = 2E−6), in the context of either TDP-43^WT^ or TDP-43^G298S^ disease models, it caused a lower turning time, indicating a rescue of locomotor dysfunction (11.30 ± 0.56 s compared to 13.40 ± 0.69 s for TDP-43^WT^ alone, *P*_value_ = 0.041; 12.50 ± 0.83 s compared to 16.40 ± 1.16 s for TDP-43^G298S^ alone, *P*_value_ = 6.4E−3, see Fig. 6a). Interestingly, although TBPH^RNAi^ knock-down also caused locomotor defects (18.75 ± 1.69 s compared to 8.95 ± 0.46 s for w^1118^, *P*_value_ = 1.66E−07), *dlp* overexpression in this context is not sufficient to mitigate TBPH^RNAi^ induced locomotor dysfunction (16.13 ± 1.72 s compared to 18.75 ± 1.69 s for TBPH^RNAi^ alone, *P*_value_ = 0.21, see Fig. 6a).

Consistent with *dlp*^*OE*^ mitigating TDP-43 induced locomotor defects, *dlp* knockdown by RNA interference (*dlp*^*RNAi*^) enhanced locomotor deficits in both ALS models based on TDP-43 overexpression (16.9 ± 1.21 s for TDP-43^WT^ dlp^RNAi^ compared to 14.00 ± 0.76 s for TDP-43^WT^ alone, *P*_value_ = 0.017; 29.30 ± 2.56 s for TDP-43^G298S^ dlp^RNAi^ compared to 16.10 ± 0.73 s for TDP-43^G298S^ alone, *P*_value_ = 1.9E−4, see Fig. 6b). In contrast, dlp^RNAi^ did not modify TBPH^RNAi^ induced locomotor dysfunction (15.6 ± 1.00 s for TBPH^RNAi^, dlp^RNAi^ compared to 17.90 ± 1.15 s for TBPH^RNAi^ alone, *P*_value_ = 0.219, see Fig. 6b), suggesting that locomotor defects caused by TBPH^RNAi^ are likely due to a different mechanism than those caused by TDP-43 overexpression, which appear to be mediated at least in part by Dlp. Surprisingly, *dlp*^*RNAi*^ knock-down caused higher larval turning times on its own (17.5 ± 0.76 s compared to 8.64 ± 0.45 s for attp40 controls, *P*_value_ = 1.4E−5, Fig. 6b) similar to *dlp*^*OE*^. These data suggest that finely tuned levels of Dlp in motor neurons are required for proper locomotor function.

We next asked whether the rescue of TDP-43 induced locomotor dysfunction by *dlp*^*OE*^ could be explained by restoration of Dlp protein levels at the NMJ. To address this, we evaluated Dlp levels within synaptic boutons when *dlp* was overexpressed on its own, in a w^1118^ background (*dlp*^*OE*^) or co-overexpressed with TDP-43^WT^, TDP-43^G298S^ or TBPH^RNAi^ (Fig. 6c-n). First we noticed that *dlp*^*OE*^ on its own caused a visible increase (3.88 times higher, *P*_value_ = 1.654E−6) in Dlp levels at the NMJ compared to w^1118^ controls (Additional file 5: Figure S6-1a-g) therefore all samples with *dlp*^OE^ were imaged at a lower gain than those without, in order to avoid saturation. While this prevented us from quantitatively comparing samples with and without *dlp*^*OE*^, we were able to evaluate Dlp levels within each set of samples (Figs. 5, 6). These experiments show that Dlp levels in terminal boutons are equal to, or greater than those observed in the *dlp*^*OE*^ alone (TDP-43^WT^ *dlp*^*OE*^ FoldChange = 2.20, *P*_value_ = 0.0432; TDP-43^G298S^ *dlp*^*OE*^, FoldChange = 1.80, *P*_value_ = 0.309; TBPH^RNAi^ *dlp*^*OE*^, FoldChange = 0.646, *P*_value_ = 1.00). Together with our findings that *dlp*^*OE*^ mitigates TDP-43 proteinopathy but not TBPH^RNAi^ induced locomotor defects, these results indicate that Dlp levels at the NMJ are critical in the context of TDP-43 proteinopathy but not TDP-43 loss, and support a cytoplasmic gain of function rather than a nuclear loss of function mechanism. It is surprising that *dlp*^*OE*^ in the context of TDP-43^WT^ causes a statistically significant increase in Dlp levels at the NMJ; this suggests a potentially complex regulatory relationship between Dlp and TDP-43 proteinopathy that warrants further investigation.

To further probe the relationship between TDP-43 induced neurodegeneration, Dlp, and Wg/Wnt signaling we used genetic interaction approaches between TDP-43 and the Frizzled 2 (Fz2) receptor, an established Dlp interactor [120]. These experiments showed that while overexpression of wild-type Fz2 had no significant effect on TDP-43 induced locomotor dysfunction (Additional file 5: Figure S6-1h), RNAi knock-down or overexpression of a dominant negative form of Fz2 caused significant larval lethality in the context of TDP-43 proteinopathy (< 10 larvae alive among > 200 total larvae, data not shown). These findings suggest that Wg/Wnt signaling may already be compromised at the NMJ in TDP-43 proteinopathy, albeit the precise mechanism remains to be determined.

### The expression of GPC6, a human Dlp ortholog is altered in patient spinal cords

**Fig. 7:**
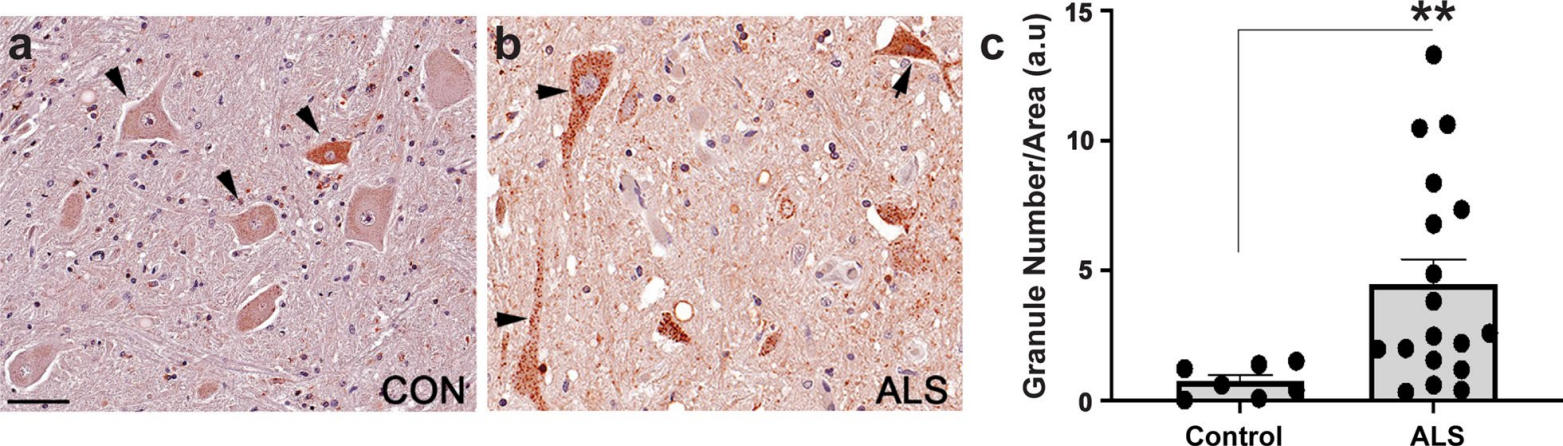
GPC6, a Dlp ortholog exhibits increased expression and altered localization in patient spinal tissues. **a** Representative image of a control spinal cord (case number CW01-72). N = 7. **b** Representative image of ALS patient spinal cord. (GWF15-07). N = 16. **c** Quantification of GPC6 granule number in post-mortem spinal tissue. Arrowheads indicate motor neurons. Significance determined using the Mann *U* Whitney Test. *P_value_ < 0.05, Scale bar in **a** 40 μm. Error bars represent SEM

To validate our findings from *Drosophila* in human patient derived tissues, we evaluated the expression and sub-cellular distribution of the Dlp human ortholog GPC6 [41] in lumbar spinal cord from ALS patients compared to non-neurological controls using immunohistochemistry. These experiments showed that GPC6 appears increased and more granular in ALS spinal cords compared to controls (Fig. 7, Additional file 4: Figure S7-1, for patient demographic information see Additional file 4: Table S7-1). Quantification of granule numbers showed that significantly more aggregate-like GPC6 puncta were present in ALS patient motor neurons compared to controls (Fold Change = 6.56, *P*_value_ = 0.0020, see Fig. 7c). Interestingly, the increased granular appearance of GPC6 in ALS spinal cords resembles the Dlp protein levels and localization observed in the *Drosophila* VNCs overexpressing TDP-43 (Fig. 5n–z). Additionally, similar to our findings in the fly models of TDP-43 proteinopathy (Fig. 4d, e), soluble vs insoluble fractionations of post-mortem patient tissue revealed that the mRNAs of both *dlp* orthologs *GPC4* and *GPC6* were enriched in the insoluble fraction of spinal cords with TDP-43 pathology relative to the insoluble fraction of occipital lobe control tissue from the same patients (N = 4 patients, *GPC4* Log2FC = 2.55,* P*_adj_ = 9.62E−17; *GPC6* Log2FC = 1.06, *P*_adj_ = 0.0122, Additional file 5: Table S7-2). Together, these findings show that GPC4/6 alterations in patient tissues resemble those identified in the *Drosophila* models of TDP-43 proteinopathy and suggest that glypican function may also be altered at neuromuscular synapses in ALS, consistent with a recent report of GPC4/6 reduction in SOD1 mice [7].

## Discussion

TDP-43 proteinopathy is a hallmark of ALS [59, 78] and has been observed in several other neurodegenerative diseases [10, 56, 59, 71, 90], yet its contribution to neurodegeneration remains poorly understood. TDP-43 contributes to multiple RNA processing steps from splicing to translational regulation, providing multiple opportunities for gene expression dysregulation in disease [30, 82]. Furthermore, TDP-43 associates with several cytoplasmic complexes including translational machinery [16, 27, 88, 92] and stress granules [13, 20, 60, 72], suggesting a role for TDP-43 in translation inhibition, as predicted by the ribostasis hypothesis [83]. Several specific mRNA targets of TDP-43 mediated translational inhibition have been reported [14, 15, 67, 68]. Among these, *futsch/MAP1B* and *Hsc70-4/HSPA8* modify disease phenotypes highlighting their contribution to disease [14, 15]. Recently, puromycin incorporation experiments in SH-SY5Y neuroblastoma cells showed that increased cytoplasmic TDP-43 reduces global translation through interactions with RACK1 on polyribosomes [88]. In contrast, polysome fractionation experiments in a human cell model showed that ALS associated mutant TDP-43^A315T^ acts as a positive regulator of translation for a subset of specific mRNAs (*e.g.*, Camta1, Mig12, and Dennd4A) [76]. Taken together these findings highlight a complex role for TDP-43 in translational regulation that can act both as a translational activator and inhibitor.

Here, using a *Drosophila* model of TDP-43 proteinopathy that recapitulates key features of the human disease including locomotor dysfunction and reduced lifespan [23, 24] we identified TDP-43 dependent changes in the motor neuron translatome. These experiments identified both pathways previously associated with ALS (*e.g.*, translation, mitochondrial function [8, 96]) and novel targets of TDP-43 mediated translational inhibition including the Wg/Wnt signaling regulator *dlp* and the glutathione metabolism pathway. Although glutathione dysregulation has been shown to be deficient both in patient tissue [118] and patient derived neuronal cultures [17], it has not previously been identified as a direct consequence of TDP-43 pathology. Surprisingly, genes related to cytoplasmic translation are enriched in the ALS motor neuron translatomes relative to the RpL10 controls, possibly reflecting a compensatory mechanism whereby degenerating neurons upregulate global translation in response to increased cellular stress or to mitigate cytoplasmic TDP-43′s inhibitory effect on protein synthesis [88].

In addition to identifying motor neuron specific alterations to the translatome induced by TDP-43 proteinopathy, we identified *dlp* as a novel target of TDP-43. *dlp* is translated into a GPI anchored glypican that interacts with the Wg/Wnt pathway receptor Fz2, serving as both a co-factor and competitive inhibitor for substrate binding [120]. RNAi knockdown or overexpression of a dominant negative form of Fz2 resulted in lethality prior to the third instar stage in the context of both TDP-43^WT^ and TDP-43^G298S^ disease models suggesting that the Wg/Wnt signaling pathway may be compromised by TDP-43 proteinopathy and consistent with published expression studies in patient tissues [34, 35]. Although overexpression of wild-type Fz2 did not mitigate TDP-43 dependent locomotor defects as would be expected based on the lethality caused by Fz2 loss of function, it is possible that cell autonomous (*i.e.*, motor neuron) and non-cell autonomous (*i.e.*, glia, muscle) aspects of Wg/Wnt signaling [47] may be at play and confound our genetic interaction experiments. Alternatively, Wg/Wnt signaling and TDP-43 proteinopathy act in parallel pathways. Indeed, Dlp could be an effector of TDP-43 proteinopathy through interactions with multiple pathways including the hedgehog [119] and hippo [4] pathways that Dlp function has also been implicated in. Our findings that both *dlp*^*OE*^ and *dlp*^*RNAi*^ cause locomotor dysfunction suggest that Dlp levels must be exquisitely regulated in motor neurons. Indeed, Dlp overexpression mitigates but does not fully rescue TDP-43 dependent locomotor defects, perhaps due to an inability to restore wild-type Dlp levels. An alternative explanation for this result is that TDP-43 regulates additional mRNA targets.

We show that although TDP-43^WT^ and TDP-43^G298S^ both associate with *dlp* mRNA, and correlate with *dlp* depletion from ribosomes, the severity of some phenotypic alterations was variant dependent. While overexpression of *dlp* mRNA was sufficient to rescue locomotor dysfunction and Dlp protein levels were altered in the context of both TDP-43 variants, *dlp* mRNA was insolubilized only in the TDP-43^G298S^ model. Additionally, different pathways were shown to have altered translation between the TDP-43^WT^ and TDP-43^G298S^ models. Differential results have previously been observed between the TDP-43^WT^ and TDP-43^G298S^ models [14], which are likely the result of increased TDP-43^G298S^ stability [58] and granule viscosity relative to TDP-43^WT^ [36].

In both ALS models, analyses of the whole VNC, which contains motor neuron soma and neurites, show that Dlp forms distinct puncta whereas staining at the NMJ revealed a significant reduction in Dlp protein. These findings suggest that TDP-43 dependent translational inhibition may be localized to axons and NMJs, which can explain the relatively low magnitude depletion of *dlp* mRNA from ribosomes using TRAP, an approach that reflects translational changes in the entire motor neuron rather than specific compartments. It is possible that the synapse specific alteration in Dlp levels could also result from transport deficits, trapping NMJ-bound *dlp* mRNA/Dlp protein in the soma or neurites. Although our qPCR data show no change in steady state *dlp* mRNA levels, we cannot eliminate the possibility of mRNA and/or protein transport defects leading to the accumulation of Dlp in the VNC and reduction at the NMJ.

**Fig. 8:**
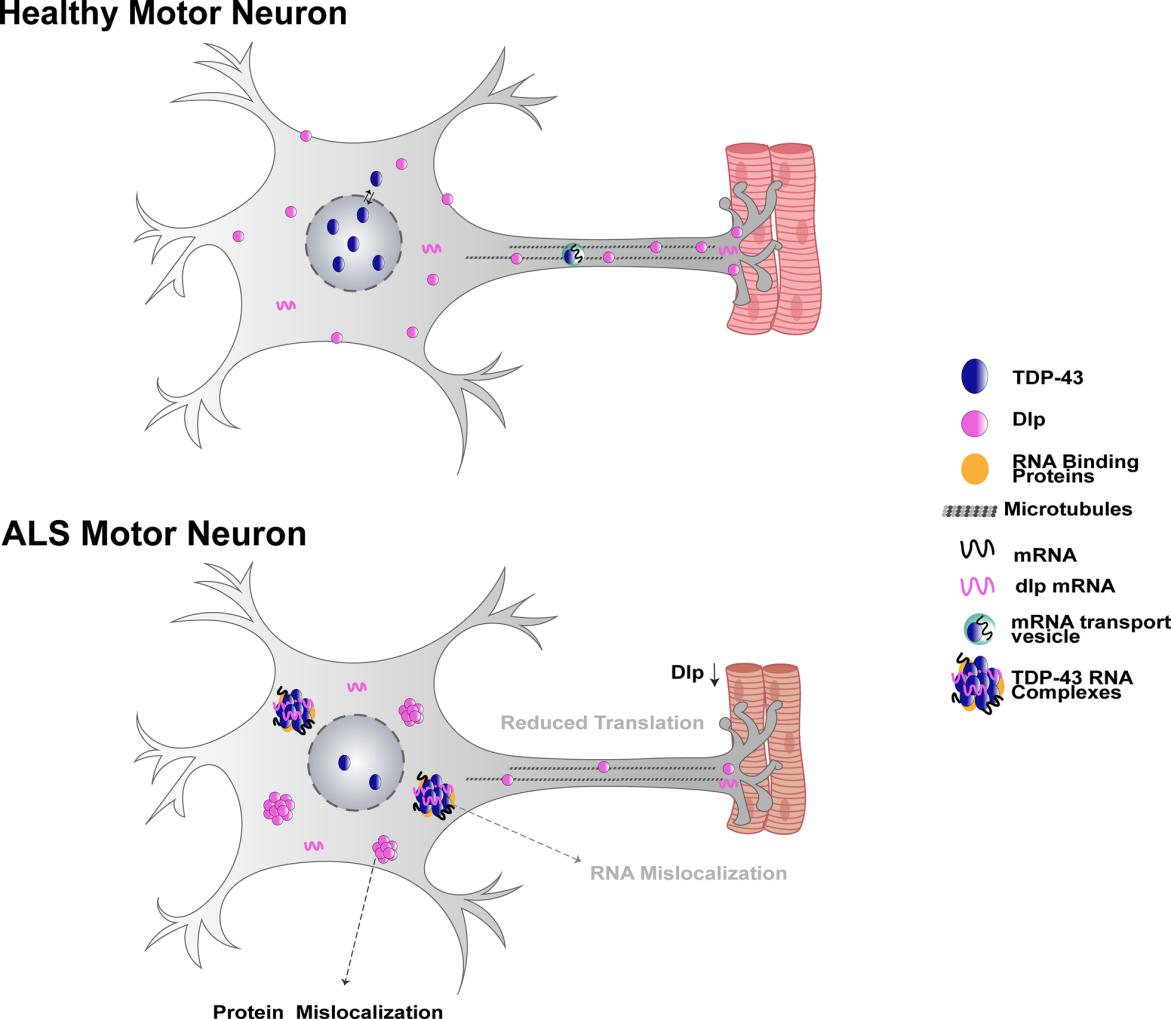
Dlp is altered in the context of TDP-43 proteinopathy. Dlp mRNA is enriched in TDP-43 complexes, depleted from ribosomes and sequestered in insoluble/urea complexes. Taken together, these findings and our observations of Dlp protein being reduced at the NMJ and accumulating in puncta within cell bodies and neuropils suggest multiple cellular defects including local translation inhibition at synapses, axonal trafficking and endomembrane trafficking deficits

Interestingly, TBPH^RNAi^ was sufficient to deplete Dlp from the NMJ, but not induce significant neuropil puncta, suggesting that the two phenotypes can occur independently of each other and might result from different mechanisms. Loss of nuclear function versus cytoplasmic gain of function remains an open question, with both mechanisms likely contributing to TDP-43 proteinopathy [53, 110]. Dlp depletion at the NMJ in the context of TBPH^RNAi^ suggests that this phenotype is the result of nuclear TDP-43 function whereas the neuropil puncta are likely the result of toxic gain of function as they are significantly increased only in the context of TDP-43 proteinopathy and not in the context of endogenous TDP-43 loss of function. Furthermore, the fact that restoring Dlp expression at the NMJ is not sufficient to mitigate TBPH^RNAi^ induced locomotor defects while it improves TDP-43^WT^ and TDP-43^G298S^ dependent phenotypes suggests that Dlp levels at the NMJ are critical in the context of TDP-43 proteinopathy and that other factors may be at play in the loss of function caused by TBPH^RNAi^ knock-down. Collectively, our findings suggest a model in which the mislocalization of Dlp found in flies and patient spinal cords results from a combination of translation inhibition and transport defects as previously reported for Futsch/MAP1B [15] (see Fig. 8 for model). Lastly, although more experiments are needed to determine the nature and composition of Dlp puncta we found in the VNC neuropil, we speculate that they may reflect a defect in intracellular trafficking and an accumulation of endomembrane compartments that could include endosomes [62], the endoplasmic reticulum [112] or the Golgi [38], which have been linked to TDP-43 proteinopathy. Identifying the subcellular compartment that these puncta associate with will be critical to understanding how Dlp misexpression relates to TDP-43 induced toxicity.

Our findings that GPC6, a Dlp ortholog, is increased in ALS spinal cord motor neurons, insolubilized in patient spinal tissues, and has puncta resembling the Dlp granules observed in the *Drosophila* VNC, further highlights the power of the fly models to predict pathological changes in disease. Interestingly, the formation of heparan sulfate proteoglycan containing puncta has previously been observed in other neurodegenerative disease including Alzheimer’s, Parkinson’s and SOD1 mediated ALS (reviewed in [66]) however this has not been previously reported in the context of TDP-43 proteinopathy, nor has a mechanism been proposed. More recently, genetic knockdown of heparan sulfate-modifying enzyme *hse-5* in C. elegans mitigated TDP-43 induced deficits in synaptic transmission [55], further suggesting a link between TDP-43 pathology, neurodegeneration and heparan sulfate proteoglycans such as Dlp/GPC6. Lastly, in the last year, genome wide association studies have identified GPC6 as a risk factor multiple sclerosis [80] and for Alzheimer’s in African Americans [51] further suggesting a role for GPC6 in maintaining neuronal function.

## Conclusions

Here we report several novel translational consequences of TDP-43 proteinopathy including effects on the spliceosome, metabolic pathways and the translational machinery itself. Our combined RIP and TRAP approach can distinguish between direct consequences of TDP-43 proteinopathy and compensatory changes in translation, which can inform future therapeutic strategies. Our identification of the glypican *dlp* as a target of TDP-43 proteinopathy highlights specific strategies including restoration of synaptic specific protein expression, intracellular transport as well as Wg/Wnt and other Dlp linked signaling pathways in ALS.

## Supplementary Information

**Additional file 1.** RNAs enriched in TDP-43 complexes, GO term analyses and comparison with mammalian TRIBE targets.

**Additional file 2.** RNAs associated with ribosomes in controls, RNAs with altered ribosome association in TDP-43 proteinopathy and TRAP GO term analyses.

**Additional file 3.** RIP/TRAP overlap in TDP-43 proteinopathy and GO term analyses.

**Additional file 4.** Demographic information for post-mortem patient spinal tissue.

**Additional file 5.** Supplemental information.

## Data Availability

The RNAseq datasets supporting the conclusions of the article are available in public repositories. *Drosophila* RNAseq data is submitted at the NCBI GEO database (GSE156222). Human RNAseq data is in the process of being submitted at the NCBI dbGaP database.

## Abbreviations

RIP: RNA Immunoprecipitation
TRAP: tagged ribosome affinity purification
NMJ: neuromuscular junction
VNC: ventral nerve cord
GFP: green fluorescent protein
YFP: yellow fluorescent protein
RNAi: RNA Interference
OE: overexpression
PPP: pentose phosphate pathway
ALS: amyotrophic lateral sclerosis
FTD: fronto-temporal dementia
TDP-43: TAR DNA binding protein 43
UAS: upstream activation sequence
DIp: Dally like protein
GPC4: glypican 4
GPC6: glypican 6

## Code availability

Code is available upon request. Trimming, alignment and differential analysis was conducted by Eric Alsop and figure generation was conducted by Erik Lehmkuhl using pre-existing programs.

## References

[1] Alami NH, Smith RB, Carrasco MA, Williams LA, Winborn CS, Han SS, Kiskinis E, Winborn B, Freibaum BD, Kanagaraj A (2014). Axonal transport of TDP-43 mRNA granules is impaired by ALS-causing mutations. Neuron.

[2] Alvarez-Castelao B, Schuman EM (2015). The regulation of synaptic protein turnover. J Biol Chem.

[3] Arnold ES, Ling SC, Huelga SC, Lagier-Tourenne C, Polymenidou M, Ditsworth D, Kordasiewicz HB, McAlonis-Downes M, Platoshyn O, Parone PA (2013). ALS-linked TDP-43 mutations produce aberrant RNA splicing and adult-onset motor neuron disease without aggregation or loss of nuclear TDP-43. Proc Natl Acad Sci USA.

[4] Baena-Lopez LA, Rodriguez I, Baonza A (2008). The tumor suppressor genes dachsous and fat modulate different signalling pathways by regulating Dally and Dally-like. Proc Natl Acad Sci USA.

[5] Bakkar N, Kovalik T, Lorenzini I, Spangler S, Lacoste A, Sponaugle K, Ferrante P, Argentinis E, Sattler R, Bowser R (2018). Artificial intelligence in neurodegenerative disease research: use of IBM Watson to identify additional RNA-binding proteins altered in amyotrophic lateral sclerosis. Acta Neuropathol.

[6] Barmada SJ, Serio A, Arjun A, Bilican B, Daub A, Ando DM, Tsvetkov A, Pleiss M, Li X, Peisach D (2014). Autophagy induction enhances TDP43 turnover and survival in neuronal ALS models. Nat Chem Biol.

[7] Cave C, Park S, Rodriguez M, Nakamura M, Hoke A, Pletnikov M, Sockanathan S (2017). GDE2 is essential for neuronal survival in the postnatal mammalian spinal cord. Mol Neurodegener.

[8] Cestra G, Rossi S, Di Salvio M, Cozzolino M (2017). Control of mRNA translation in ALS proteinopathy. Front Mol Neurosci.

[9] Chang JC, Morton DB (2017). Drosophila lines with mutant and wild type human TDP-43 replacing the endogenous gene reveals phosphorylation and ubiquitination in mutant lines in the absence of viability or lifespan defects. PLoS ONE.

[10] Chang XL, Tan MS, Tan L, Yu JT (2016). The role of TDP-43 in Alzheimer's disease. Mol Neurobiol.

[11] Chen Y, Guan Y, Zhang Z, Liu H, Wang S, Yu L, Wu X, Wang X (2012). Wnt signaling pathway is involved in the pathogenesis of amyotrophic lateral sclerosis in adult transgenic mice. Neurol Res.

[12] Chu JF, Majumder P, Chatterjee B, Huang SL, Shen CJ (2019). TDP-43 regulates coupled dendritic mRNA transport-translation processes in co-operation with FMRP and Staufen1. Cell Rep.

[13] Colombrita C, Zennaro E, Fallini C, Weber M, Sommacal A, Buratti E, Silani V, Ratti A (2009). TDP-43 is recruited to stress granules in conditions of oxidative insult. J Neurochem.

[14] Coyne AN, Lorenzini I, Chou CC, Torvund M, Rogers RS, Starr A, Zaepfel BL, Levy J, Johannesmeyer J, Schwartz JC (2017). Post-transcriptional inhibition of Hsc70-4/HSPA8 expression leads to synaptic vesicle cycling defects in multiple models of ALS. Cell Rep.

[15] Coyne AN, Siddegowda BB, Estes PS, Johannesmeyer J, Kovalik T, Daniel SG, Pearson A, Bowser R, Zarnescu DC (2014). Futsch/MAP1B mRNA Is a translational target of TDP-43 and is neuroprotective in a drosophila model of amyotrophic lateral sclerosis. J Neurosci.

[16] Coyne AN, Yamada SB, Siddegowda BB, Estes PS, Zaepfel BL, Johannesmeyer JS, Lockwood DB, Pham LT, Hart MP, Cassel JA (2015). Fragile X protein mitigates TDP-43 toxicity by remodeling RNA granules and restoring translation. Hum Mol Genet.

[17] D'Alessandro G, Calcagno E, Tartari S, Rizzardini M, Invernizzi RW, Cantoni L (2011). Glutamate and glutathione interplay in a motor neuronal model of amyotrophic lateral sclerosis reveals altered energy metabolism. Neurobiol Dis.

[18] De Conti L, Akinyi MV, Mendoza-Maldonado R, Romano M, Baralle M, Buratti E (2015). TDP-43 affects splicing profiles and isoform production of genes involved in the apoptotic and mitotic cellular pathways. Nucleic Acids Res.

[19] DeJesus-Hernandez M, Mackenzie IR, Boeve BF, Boxer AL, Baker M, Rutherford NJ, Nicholson AM, Finch NA, Flynn H, Adamson J (2011). Expanded GGGGCC hexanucleotide repeat in noncoding region of C9ORF72 causes chromosome 9p-linked FTD and ALS. Neuron.

[20] Dewey CM, Cenik B, Sephton CF, Dries DR, Mayer P, Good SK, Johnson BA, Herz J, Yu G (2011). TDP-43 is directed to stress granules by sorbitol, a novel physiological osmotic and oxidative stressor. Mol Cell Biol.

[21] Dewey CM, Cenik B, Sephton CF, Johnson BA, Herz J, Yu G (2012). TDP-43 aggregation in neurodegeneration: are stress granules the key?. Brain Res.

[22] Dupuis L, Pradat PF, Ludolph AC, Loeffler JP (2011). Energy metabolism in amyotrophic lateral sclerosis. Lancet Neurol.

[23] Estes PS, Boehringer A, Zwick R, Tang JE, Grigsby B, Zarnescu DC (2011). Wild-type and A315T mutant TDP-43 exert differential neurotoxicity in a Drosophila model of ALS. Hum Mol Genet.

[24] Estes PS, Daniel SG, McCallum AP, Boehringer AV, Sukhina AS, Zwick RA, Zarnescu DC (2013). Motor neurons and glia exhibit specific individualized responses to TDP-43 expression in a Drosophila model of amyotrophic lateral sclerosis. Dis Model Mech.

[25] Fernandes N, Eshleman N, Buchan JR (2018). Stress granules and ALS: a case of causation or correlation?. Adv Neurobiol.

[26] Fiesel FC, Weber SS, Supper J, Zell A, Kahle PJ (2012). TDP-43 regulates global translational yield by splicing of exon junction complex component SKAR. Nucleic Acids Res.

[27] Freibaum BD, Chitta RK, High AA, Taylor JP (2010). Global analysis of TDP-43 interacting proteins reveals strong association with RNA splicing and translation machinery. J Proteome Res.

[28] Gamo K, Kiryu-Seo S, Konishi H, Aoki S, Matsushima K, Wada K, Kiyama H (2008). G-protein-coupled receptor screen reveals a role for chemokine receptor CCR5 in suppressing microglial neurotoxicity. J Neurosci.

[29] Gan G, Lv H, Xie W (2014). Morphological identification and development of neurite in Drosophila ventral nerve cord neuropil. PLoS ONE.

[30] Gao J, Wang L, Huntley ML, Perry G, Wang X (2018). Pathomechanisms of TDP-43 in neurodegeneration. J Neurochem.

[31] Gasset-Rosa F, Lu S, Yu H, Chen C, Melamed Z, Guo L, Shorter J, Da Cruz S, Cleveland DW (2019). Cytoplasmic TDP-43 De-mixing independent of stress granules drives inhibition of nuclear import, loss of nuclear TDP-43, and cell death. Neuron.

[32] Gil J, Funalot B, Verschueren A, Danel-Brunaud V, Camu W, Vandenberghe N, Desnuelle C, Guy N, Camdessanche JP, Cintas P (2008). Causes of death amongst French patients with amyotrophic lateral sclerosis: a prospective study. Eur J Neurol.

[33] Godena VK, Romano G, Romano M, Appocher C, Klima R, Buratti E, Baralle FE, Feiguin F (2011). TDP-43 regulates Drosophila neuromuscular junctions growth by modulating Futsch/MAP1B levels and synaptic microtubules organization. PLoS ONE.

[34] Gonzalez-Fernandez C, Gonzalez P, Andres-Benito P, Ferrer I, Rodriguez FJ (2019). Wnt signaling alterations in the human spinal cord of amyotrophic lateral sclerosis cases: spotlight on Fz2 and Wnt5a. Mol Neurobiol.

[35] Gonzalez-Fernandez C, Gonzalez P, Rodriguez FJ (2020). New insights into Wnt signaling alterations in amyotrophic lateral sclerosis: a potential therapeutic target?. Neural Regen Res.

[36] Gopal PP, Nirschl JJ, Klinman E, Holzbaur EL (2017). Amyotrophic lateral sclerosis-linked mutations increase the viscosity of liquid-like TDP-43 RNP granules in neurons. Proc Natl Acad Sci USA.

[37] Gustafson K, Boulianne GL (1996). Distinct expression patterns detected within individual tissues by the GAL4 enhancer trap technique. Genome.

[38] Haase G, Rabouille C (2015). Golgi fragmentation in ALS motor neurons. New mechanisms targeting microtubules, tethers, and transport vesicles. Front Neurosci.

[39] Heiman M, Schaefer A, Gong S, Peterson JD, Day M, Ramsey KE, Suarez-Farinas M, Schwarz C, Stephan DA, Surmeier DJ (2008). A translational profiling approach for the molecular characterization of CNS cell types. Cell.

[40] Herzog JJ, Xu W, Deshpande M, Rahman R, Suib H, Rodal AA, Rosbash M, Paradis S (2020). TDP-43 dysfunction restricts dendritic complexity by inhibiting CREB activation and altering gene expression. Proc Natl Acad Sci USA.

[41] Hu Y, Flockhart I, Vinayagam A, Bergwitz C, Berger B, Perrimon N, Mohr SE (2011). An integrative approach to ortholog prediction for disease-focused and other functional studies. BMC Bioinformatics.

[42] da Huang W, Sherman BT, Lempicki RA (2009). Bioinformatics enrichment tools: paths toward the comprehensive functional analysis of large gene lists. Nucleic Acids Res.

[43] da Huang W, Sherman BT, Lempicki RA (2009). Systematic and integrative analysis of large gene lists using DAVID bioinformatics resources. Nat Protoc.

[44] Ihara R, Matsukawa K, Nagata Y, Kunugi H, Tsuji S, Chihara T, Kuranaga E, Miura M, Wakabayashi T, Hashimoto T (2013). RNA binding mediates neurotoxicity in the transgenic Drosophila model of TDP-43 proteinopathy. Hum Mol Genet.

[45] Kabashi E, Valdmanis PN, Dion P, Spiegelman D, McConkey BJ, Vande Velde C, Bouchard JP, Lacomblez L, Pochigaeva K, Salachas F (2008). TARDBP mutations in individuals with sporadic and familial amyotrophic lateral sclerosis. Nat Genet.

[46] Khalfallah Y, Kuta R, Grasmuck C, Prat A, Durham HD, Vande Velde C (2018). TDP-43 regulation of stress granule dynamics in neurodegenerative disease-relevant cell types. Sci Rep.

[47] Koles K, Budnik V (2012). Wnt signaling in neuromuscular junction development. Cold Spring Harb Perspect Biol.

[48] Krach F, Batra R, Wheeler EC, Vu AQ, Wang R, Hutt K, Rabin SJ, Baughn MW, Libby RT, Diaz-Garcia S (2018). Transcriptome-pathology correlation identifies interplay between TDP-43 and the expression of its kinase CK1E in sporadic ALS. Acta Neuropathol.

[49] Krug L, Chatterjee N, Borges-Monroy R, Hearn S, Liao WW, Morrill K, Prazak L, Rozhkov N, Theodorou D, Hammell M (2017). Retrotransposon activation contributes to neurodegeneration in a Drosophila TDP-43 model of ALS. PLoS Genet.

[50] Kruger NJ, von Schaewen A (2003). The oxidative pentose phosphate pathway: structure and organisation. Curr Opin Plant Biol.

[51] Kunkle BW, Schmidt M, Klein HU, Naj AC, Hamilton-Nelson KL, Larson EB, Evans DA, De Jager PL, Crane PK, Buxbaum JD (2021). Novel Alzheimer disease risk loci and pathways in African American individuals using the african genome resources panel: a meta-analysis. JAMA Neurol.

[52] Lattante S, Ciura S, Rouleau GA, Kabashi E (2015). Defining the genetic connection linking amyotrophic lateral sclerosis (ALS) with frontotemporal dementia (FTD). Trends Genet.

[53] Lee EB, Lee VM, Trojanowski JQ (2011). Gains or losses: molecular mechanisms of TDP43-mediated neurodegeneration. Nat Rev Neurosci.

[54] Legan SK, Rebrin I, Mockett RJ, Radyuk SN, Klichko VI, Sohal RS, Orr WC (2008). Overexpression of glucose-6-phosphate dehydrogenase extends the life span of Drosophila melanogaster. J Biol Chem.

[55] Liachko NF, Saxton AD, McMillan PJ, Strovas TJ, Keene CD, Bird TD, Kraemer BC (2019). Genome wide analysis reveals heparan sulfate epimerase modulates TDP-43 proteinopathy. PLoS Genet.

[56] Ling H, Hardy J, Zetterberg H (2015). Neurological consequences of traumatic brain injuries in sports. Mol Cell Neurosci.

[57] Ling JP, Pletnikova O, Troncoso JC, Wong PC (2015). TDP-43 repression of nonconserved cryptic exons is compromised in ALS-FTD. Science.

[58] Ling SC, Albuquerque CP, Han JS, Lagier-Tourenne C, Tokunaga S, Zhou H, Cleveland DW (2010). ALS-associated mutations in TDP-43 increase its stability and promote TDP-43 complexes with FUS/TLS. Proc Natl Acad Sci USA.

[59] Ling SC, Polymenidou M, Cleveland DW (2013). Converging mechanisms in ALS and FTD: disrupted RNA and protein homeostasis. Neuron.

[60] Liu-Yesucevitz L, Bilgutay A, Zhang YJ, Vanderweyde T, Citro A, Mehta T, Zaarur N, McKee A, Bowser R, Sherman M (2010). Tar DNA binding protein-43 (TDP-43) associates with stress granules: analysis of cultured cells and pathological brain tissue. PLoS ONE.

[61] Liu EY, Russ J, Cali CP, Phan JM, Amlie-Wolf A, Lee EB (2019). Loss of nuclear TDP-43 is associated with decondensation of LINE retrotransposons. Cell Rep.

[62] Liu G, Coyne AN, Pei F, Vaughan S, Chaung M, Zarnescu DC, Buchan JR (2017). Endocytosis regulates TDP-43 toxicity and turnover. Nat Commun.

[63] Liu J, Wang F (2017). Role of neuroinflammation in amyotrophic lateral sclerosis: cellular mechanisms and therapeutic implications. Front Immunol.

[64] Love MI, Huber W, Anders S (2014). Moderated estimation of fold change and dispersion for RNA-seq data with DESeq2. Genome Biol.

[65] MacNair L, Xiao S, Miletic D, Ghani M, Julien JP, Keith J, Zinman L, Rogaeva E, Robertson J (2016). MTHFSD and DDX58 are novel RNA-binding proteins abnormally regulated in amyotrophic lateral sclerosis. Brain.

[66] Maiza A, Chantepie S, Vera C, Fifre A, Huynh MB, Stettler O, Ouidja MO, Papy-Garcia D (2018). The role of heparan sulfates in protein aggregation and their potential impact on neurodegeneration. FEBS Lett.

[67] Majumder P, Chen YT, Bose JK, Wu CC, Cheng WC, Cheng SJ, Fang YH, Chen YL, Tsai KJ, Lien CC (2012). TDP-43 regulates the mammalian spinogenesis through translational repression of Rac1. Acta Neuropathol.

[68] Majumder P, Chu JF, Chatterjee B, Swamy KB, Shen CJ (2016). Co-regulation of mRNA translation by TDP-43 and Fragile X syndrome protein FMRP. Acta Neuropathol.

[69] Mann JR, Gleixner AM, Mauna JC, Gomes E, DeChellis-Marks MR, Needham PG, Copley KE, Hurtle B, Portz B, Pyles NJ (2019). RNA binding antagonizes neurotoxic phase transitions of TDP-43. Neuron.

[70] Manzo E, Lorenzini I, Barrameda D, O'Conner AG, Barrows JM, Starr A, Kovalik T, Rabichow BE, Lehmkuhl EM, Shreiner DD (2019). Glycolysis upregulation is neuroprotective as a compensatory mechanism in ALS. eLife.

[71] Markopoulou K, Dickson DW, McComb RD, Wszolek ZK, Katechalidou L, Avery L, Stansbury MS, Chase BA (2008). Clinical, neuropathological and genotypic variability in SNCA A53T familial Parkinson's disease. Variability in familial Parkinson's disease. Acta Neuropathol.

[72] McDonald KK, Aulas A, Destroismaisons L, Pickles S, Beleac E, Camu W, Rouleau GA, Vande Velde C (2011). TAR DNA-binding protein 43 (TDP-43) regulates stress granule dynamics via differential regulation of G3BP and TIA-1. Hum Mol Genet.

[73] McQuin C, Goodman A, Chernyshev V, Kamentsky L, Cimini BA, Karhohs KW, Doan M, Ding L, Rafelski SM, Thirstrup D (2018). Cell Profiler 3.0: next-generation image processing for biology. PLoS Biol.

[74] Menon P, Higashihara M, van den Bos M, Geevasinga N, Kiernan MC, Vucic S (2020). Cortical hyperexcitability evolves with disease progression in ALS. Ann Clin Transl Neurol.

[75] Morera AA, Ahmed NS, Schwartz JC (2019). TDP-43 regulates transcription at protein-coding genes and Alu retrotransposons. Biochim Biophys Acta Gene Regul Mech.

[76] Neelagandan N, Gonnella G, Dang S, Janiesch PC, Miller KK, Kuchler K, Marques RF, Indenbirken D, Alawi M, Grundhoff A (2019). TDP-43 enhances translation of specific mRNAs linked to neurodegenerative disease. Nucleic Acids Res.

[77] Neumann M (2009). Molecular neuropathology of TDP-43 proteinopathies. Int J Mol Sci.

[78] Neumann M, Sampathu DM, Kwong LK, Truax AC, Micsenyi MC, Chou TT, Bruce J, Schuck T, Grossman M, Clark CM (2006). Ubiquitinated TDP-43 in frontotemporal lobar degeneration and amyotrophic lateral sclerosis. Science.

[79] Niedermeyer S, Murn M, Choi PJ (2019). Respiratory failure in amyotrophic lateral sclerosis. Chest.

[80] Okolicsanyi RK, Bluhm J, Miller C, Griffiths LR, Haupt LM (2020). An investigation of genetic polymorphisms in heparan sulfate proteoglycan core proteins and key modification enzymes in an Australian Caucasian multiple sclerosis population. Hum Genom.

[81] Pfaffl MW (2001). A new mathematical model for relative quantification in real-time RT-PCR. Nucleic Acids Res.

[82] Prasad A, Bharathi V, Sivalingam V, Girdhar A, Patel BK (2019). Molecular mechanisms of TDP-43 misfolding and pathology in amyotrophic lateral sclerosis. Front Mol Neurosci.

[83] Ramaswami M, Taylor JP, Parker R (2013). Altered ribostasis: RNA-protein granules in degenerative disorders. Cell.

[84] Renton AE, Majounie E, Waite A, Simon-Sanchez J, Rollinson S, Gibbs JR, Schymick JC, Laaksovirta H, van Swieten JC, Myllykangas L (2011). A hexanucleotide repeat expansion in C9ORF72 is the cause of chromosome 9p21-linked ALS-FTD. Neuron.

[85] Rio DC, Ares M, Hannon GJ, Nilsen TW (2010). Purification of RNA using TRIzol (TRI reagent). Cold Spring Harb Protoc.

[86] Ritson GP, Custer SK, Freibaum BD, Guinto JB, Geffel D, Moore J, Tang W, Winton MJ, Neumann M, Trojanowski JQ (2010). TDP-43 mediates degeneration in a novel Drosophila model of disease caused by mutations in VCP/p97. J Neurosci.

[87] Rosen DR, Siddique T, Patterson D, Figlewicz DA, Sapp P, Hentati A, Donaldson D, Goto J, O'Regan JP, Deng HX (1993). Mutations in Cu/Zn superoxide dismutase gene are associated with familial amyotrophic lateral sclerosis. Nature.

[88] Russo A, Scardigli R, La Regina F, Murray ME, Romano N, Dickson DW, Wolozin B, Cattaneo A, Ceci M (2017). Increased cytoplasmic TDP-43 reduces global protein synthesis by interacting with RACK1 on polyribosomes. Hum Mol Genet.

[89] Salajegheh M, Pinkus JL, Taylor JP, Amato AA, Nazareno R, Baloh RH, Greenberg SA (2009). Sarcoplasmic redistribution of nuclear TDP-43 in inclusion body myositis. Muscle Nerve.

[90] Schwab C, Arai T, Hasegawa M, Yu S, McGeer PL (2008). Colocalization of transactivation-responsive DNA-binding protein 43 and huntingtin in inclusions of Huntington disease. J Neuropathol Exp Neurol.

[91] Sephton CF, Cenik B, Cenik BK, Herz J, Yu G (2012). TDP-43 in central nervous system development and function: clues to TDP-43-associated neurodegeneration. Biol Chem.

[92] Sephton CF, Cenik C, Kucukural A, Dammer EB, Cenik B, Han Y, Dewey CM, Roth FP, Herz J, Peng J (2011). Identification of neuronal RNA targets of TDP-43-containing ribonucleoprotein complexes. J Biol Chem.

[93] Shenouda M, Zhang AB, Weichert A, Robertson J (2018). Mechanisms associated with TDP-43 neurotoxicity in ALS/FTLD. Adv Neurobiol.

[94] Shulman RG, Rothman DL, Behar KL, Hyder F (2004). Energetic basis of brain activity: implications for neuroimaging. Trends Neurosci.

[95] Singh A, Kukreti R, Saso L, Kukreti S (2019). Oxidative stress: a key modulator in neurodegenerative diseases. Molecules.

[96] Smith EF, Shaw PJ, De Vos KJ (2019). The role of mitochondria in amyotrophic lateral sclerosis. Neurosci Lett.

[97] Spampinato SF, Copani A, Nicoletti F, Sortino MA, Caraci F (2018). Metabotropic glutamate receptors in glial cells: a new potential target for neuroprotection?. Front Mol Neurosci.

[98] Sreedharan J, Blair IP, Tripathi VB, Hu X, Vance C, Rogelj B, Ackerley S, Durnall JC, Williams KL, Buratti E (2008). TDP-43 mutations in familial and sporadic amyotrophic lateral sclerosis. Science.

[99] Strah N, Romano G, Introna C, Klima R, Marzullo M, Ciapponi L, Megighian A, Nizzardo M, Feiguin F (2020). TDP-43 promotes the formation of neuromuscular synapses through the regulation of Disc-large expression in Drosophila skeletal muscles. BMC Biol.

[100] Stryker E, Johnson KG (2007). LAR, liprin alpha and the regulation of active zone morphogenesis. J Cell Sci.

[101] Swain A, Misulovin Z, Pherson M, Gause M, Mihindukulasuriya K, Rickels RA, Shilatifard A, Dorsett D (2016). Drosophila TDP-43 RNA-binding protein facilitates association of sister chromatid cohesion proteins with genes, enhancers and polycomb response elements. PLoS Genet.

[102] Szklarczyk D, Gable AL, Lyon D, Junge A, Wyder S, Huerta-Cepas J, Simonovic M, Doncheva NT, Morris JH, Bork P (2019). STRING v11: protein-protein association networks with increased coverage, supporting functional discovery in genome-wide experimental datasets. Nucleic Acids Res.

[103] Tam OH, Rozhkov NV, Shaw R, Kim D, Hubbard I, Fennessey S, Propp N, Consortium NA, Fagegaltier D, Harris BT (2019). Postmortem cortex samples identify distinct molecular subtypes of ALS: retrotransposon activation, oxidative stress, and activated glia. Cell Rep.

[104] Tank EM, Figueroa-Romero C, Hinder LM, Bedi K, Archbold HC, Li X, Weskamp K, Safren N, Paez-Colasante X, Pacut C (2018). Abnormal RNA stability in amyotrophic lateral sclerosis. Nat Commun.

[105] Taylor JP, Brown RH, Cleveland DW (2016). Decoding ALS: from genes to mechanism. Nature.

[106] Tefera TW, Borges K (2016). Metabolic dysfunctions in amyotrophic lateral sclerosis pathogenesis and potential metabolic treatments. Front Neurosci.

[107] Thomas A, Lee PJ, Dalton JE, Nomie KJ, Stoica L, Costa-Mattioli M, Chang P, Nuzhdin S, Arbeitman MN, Dierick HA (2012). A versatile method for cell-specific profiling of translated mRNAs in Drosophila. PLoS ONE.

[108] Tollervey JR, Curk T, Rogelj B, Briese M, Cereda M, Kayikci M, Konig J, Hortobagyi T, Nishimura AL, Zupunski V (2011). Characterizing the RNA targets and position-dependent splicing regulation by TDP-43. Nat Neurosci.

[109] Van Deerlin VM, Leverenz JB, Bekris LM, Bird TD, Yuan W, Elman LB, Clay D, Wood EM, Chen-Plotkin AS, Martinez-Lage M (2008). TARDBP mutations in amyotrophic lateral sclerosis with TDP-43 neuropathology: a genetic and histopathological analysis. Lancet Neurol.

[110] Vanden Broeck L, Callaerts P, Dermaut B (2014). TDP-43-mediated neurodegeneration: towards a loss-of-function hypothesis?. Trends Mol Med.

[111] Vogler TO, Wheeler JR, Nguyen ED, Hughes MP, Britson KA, Lester E, Rao B, Betta ND, Whitney ON, Ewachiw TE (2018). TDP-43 and RNA form amyloid-like myo-granules in regenerating muscle. Nature.

[112] Walker AK, Soo KY, Sundaramoorthy V, Parakh S, Ma Y, Farg MA, Wallace RH, Crouch PJ, Turner BJ, Horne MK (2013). ALS-associated TDP-43 induces endoplasmic reticulum stress, which drives cytoplasmic TDP-43 accumulation and stress granule formation. PLoS ONE.

[113] Walker AK, Spiller KJ, Ge G, Zheng A, Xu Y, Zhou M, Tripathy K, Kwong LK, Trojanowski JQ, Lee VM (2015). Functional recovery in new mouse models of ALS/FTLD after clearance of pathological cytoplasmic TDP-43. Acta Neuropathol.

[114] Walter W, Sanchez-Cabo F, Ricote M (2015). GOplot: an R package for visually combining expression data with functional analysis. Bioinformatics.

[115] Wang F, Flanagan J, Su N, Wang LC, Bui S, Nielson A, Wu X, Vo HT, Ma XJ, Luo Y (2012). RNAscope: a novel in situ RNA analysis platform for formalin-fixed, paraffin-embedded tissues. J Mol Diagn.

[116] Wang IF, Guo BS, Liu YC, Wu CC, Yang CH, Tsai KJ, Shen CK (2012). Autophagy activators rescue and alleviate pathogenesis of a mouse model with proteinopathies of the TAR DNA-binding protein 43. Proc Natl Acad Sci USA.

[117] Wang P, Deng J, Dong J, Liu J, Bigio EH, Mesulam M, Wang T, Sun L, Wang L, Lee AY (2019). TDP-43 induces mitochondrial damage and activates the mitochondrial unfolded protein response. PLoS Genet.

[118] Weiduschat N, Mao X, Hupf J, Armstrong N, Kang G, Lange DJ, Mitsumoto H, Shungu DC (2014). Motor cortex glutathione deficit in ALS measured in vivo with the J-editing technique. Neurosci Lett.

[119] Williams EH, Pappano WN, Saunders AM, Kim MS, Leahy DJ, Beachy PA (2010). Dally-like core protein and its mammalian homologues mediate stimulatory and inhibitory effects on Hedgehog signal response. Proc Natl Acad Sci U S A.

[120] Yan D, Wu Y, Feng Y, Lin SC, Lin X (2009). The core protein of glypican Dally-like determines its biphasic activity in wingless morphogen signaling. Dev Cell.

