# Supplementary material for: TDP-43 proteinopathy alters the ribosome association of multiple mRNAs including the glypican Dally-like protein (Dlp)/GPC6
Source: Acta Neuropathol Commun. 2021 Mar 24;9:52. doi: 10.1186/s40478-021-01148-z (PMC7992842; doi:10.1186/s40478-021-01148-z)
Supplement: Supplementary file 5 — Additional file 5. Supplemental information. [file 40478_2021_1148_MOESM5_ESM.docx]

**­­­­­
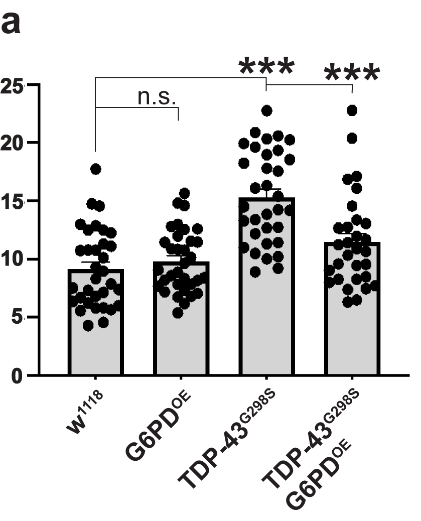
**

**Supplemental Figure 2-1. Increased Pentose Phosphate Pathway Activity Rescues TDP-43^G298S^ Induced Locomotor Dysfunction. (a).** Larval turning times for *G6PD* mRNA overexpression in the w^1118^ genetic background (control for OE experiments) and TDP-43^G298S^.. N = 33 for w^1118^, 33 for G6PD^OE^, 34 for TDP-43^G298S^, 30 for TDP-43^G298S^ G6PD^OE^). P_value_ < 0.05, ** = P_value_ < 0.01, *** = P_value_ < 0.001. Error bars represent SEM.

**
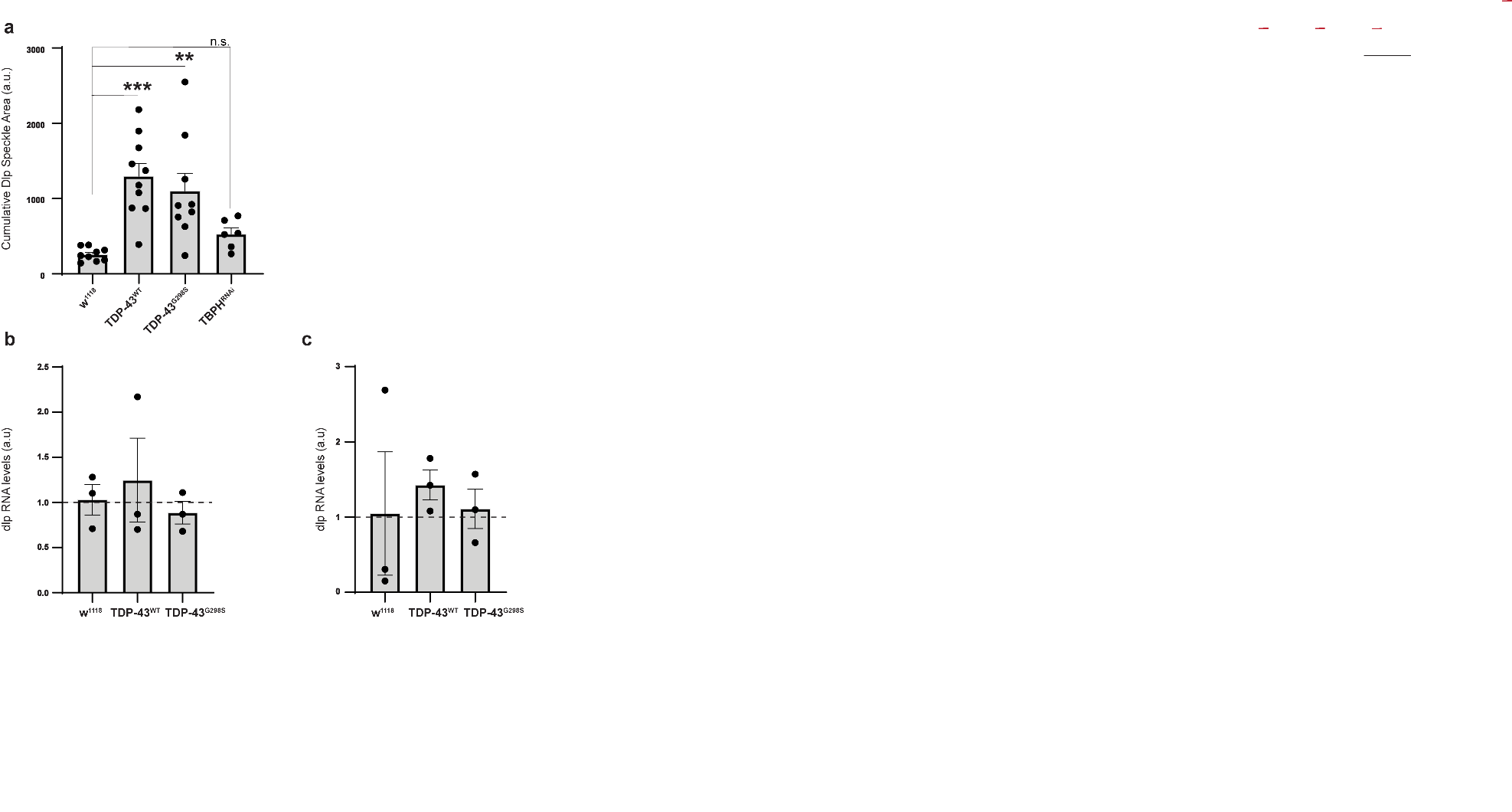
**

**Supplemental Figure 5-1. Alterations to *dlp* RNA levels were not detected in either the NMJ or the whole VNC (a).** Cumulative speckle area of Dlp granules in the VNC neuropil from figure 5. N = 9 for w^1118^, 10 for TDP-43^WT^, 9 for TDP-43^G298S^, 6 for TBPH^RNAi^. **(b).** Quantification of *dlp* mRNA relative to *GPDH* in dissected VNCs. N = 3. (**c).** Quantification of *dlp* mRNA relative to *GPDH* in dissected NMJs. N = 3. Significance determined through Holm Sidak’s multiple comparison test. * = Pvalue < 0.05, ** = Pvalue < 0.01, *** = Pvalue < 0.001. Error bars represent SEM.

**
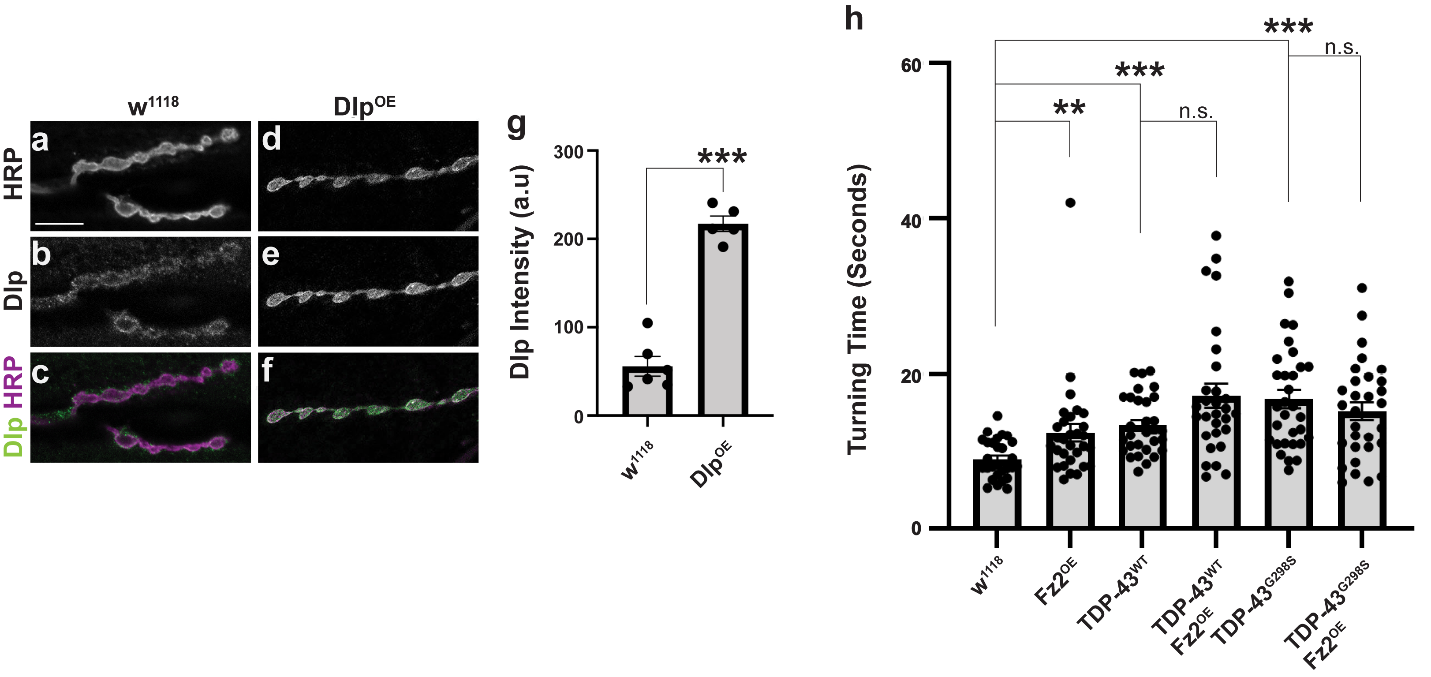
**

**Supplemental Figure 6-1. Cumulative Dlp speckle area in the context of TBPH-RNAi. (a-f)** Representative images of w^1118^ and Dlp^OE^ NMJs stained for Dlp and HRP. **(g).** Quantification of Dlp granule number in the neuropil. N = (w^1118^ = 6, dlp^OE^ = 5). Genotypes and stainings, as indicated. Scale bars: **(a)** – 10 um. Significance determined through student’s T Test. (t_value_ = 10.96, degrees of freedom = 9). * = Pvalue < 0.05, ** = Pvalue < 0.01, *** = Pvalue < 0.001 **(h)** Larval turning times for *Fz2* mRNA overexpression in the w^1118^ genetic background (control for OE experiments), TDP-43^WT^, and TDP-43^G298S^. N = 29 for w^1118^, 30 for Fz2^OE^, 30 for TDP-43^WT^, 29 for TDP-43^WT^ Fz2^OE^, 31 for TDP-43^G298S^, 30 for TDP-43^G298S^ Fz2^OE^. Significance determined using the MannU Whitney Test or Holm Sidak’s multiple comparison test. * = Pvalue < 0.05, ** = Pvalue < 0.01, *** = Pvalue < 0.001. Error bars represent SEM.


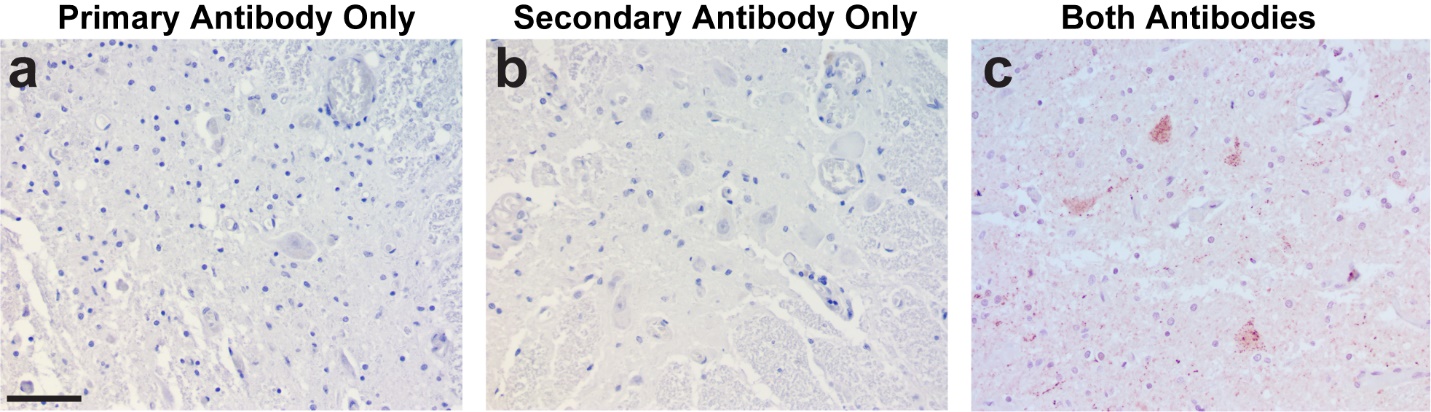


**Supplemental Figure 7-1. GPC6 Staining – Single Antibody Controls. (a-c)** All images are from case GWF15-11. **(a)** Spinal cord treated with only primary antibody (anti-GPC6 1:600, Bioss bs-2177R). **(b)** Spinal cord treated with only secondary antibody (anti-rabbit 1:200, Vector Laboratories BA-1000). **(c)** Spinal cord treated with primary and secondary antibodies to detect GPC6. Scale bar in **(a)** 20 μm.

Figure 4 – Supplemental Table 1. *dlp* enrichment with TDP-43 (RIP) and depletion from the ribosome in ALS models relative to the RPL10 control (TRAP).

|  | RIP TDP-43WT | | RIP TDP-43--G298S | | TRAP TDP-43-WT | | TRAP TDP-43-G298S | |
| --- | --- | --- | --- | --- | --- | --- | --- | --- |
| Gene | Log2FC | Padj. | Log2FC | Padj. | Log2FC | Padj. | Log2FC | Padj. |
| *dlp* | 3.62 | 8.32E-57 | 4.63 | 2.47E-57 | -0.394 | 0.0274 | -0.213 | 0.0121 |

Figure 7 – Supplemental Table 2. *GPC4/GPC6* differential expression analysis comparing the urea fractionation of patient spinal tissue containing TDP-43 pathology to the urea fractionation of control tissue.

| Ensembl Gene ID | baseMean | log2FoldChange | lfcSE | stat | pvalue | padj | Official Gene Symbol |
| --- | --- | --- | --- | --- | --- | --- | --- |
| ENSG00000076716 | 1678.521 | 2.551 | 0.293748 | 8.684306 | 3.81E-18 | 9.62E-17 | GPC4 |
| ENSG00000183098 | 850.7872 | 1.059793 | 0.3676 | 2.883005 | 0.003939 | 0.012263 | GPC6 |
